# Eight new species of the spider genera *Raveniola* and *Sinopesa* from China and Vietnam (Araneae, Nemesiidae)

**DOI:** 10.3897/zookeys.519.8784

**Published:** 2015-08-26

**Authors:** Shuqiang Li, Sergei Zonstein

**Affiliations:** 1 Institute of Zoology, Chinese Academy of Sciences, Beijing 100101, China; 2 Department of Zoology, The George S. Wise Faculty of Life Sciences, Tel-Aviv University, 69978 Tel-Aviv, Israel

**Keywords:** Taxonomy, mygalomorph spiders, new records, South-East Asia

## Abstract

Eight new species, seven *Raveniola* Zonstein, 1987 and one *Sinopesa* Raven & Schwendinger, 1995 from China and Vietnam are described: *Raveniola alpina* **sp. n.**, *Raveniola bellula* **sp. n.**, *Raveniola chayi* **sp. n.**, *Raveniola gracilis* **sp. n.**, *Raveniola rugosa* **sp. n.**, *Raveniola spirula* **sp. n.** and *Raveniola yajiangensis* **sp. n.** and *Sinopesa ninhbinhensis* **sp. n.** Keys to all East-Asian congeners, diagnoses of the new species, and new distribution data of *Raveniola montana* Zonstein & Marusik, 2012, with a first record for Sichuan, China, are provided.

## Introduction

The Nemesiidae are known to include 45 genera and 382 species (World Spider Catalog 2015). The members of five nemesiid genera are distributed within Eastern and South-Eastern Asia: *Atmetochilus* Simon, 1887, *Damarchilus* Silival, Molur & Raven, 2015, *Damarchus* Thorell, 1891, *Raveniola* Zonstein, 1987 and *Sinopesa* Raven & Schwendinger, 1995. Before our study, eleven species in three genera were known from China (Li and Wang 2014; Zonstein and Marusik 2012) and none from Vietnam.

While sorting and identifying nemesiid material in the Institute of Zoology, Chinese Academy of Sciences, eight new species belonging to *Raveniola* and *Sinopesa* were found; they are described here. The systematic position of both genera was recently considered by Zonstein and Marusik (2012). The latter genus was shown to be more closely related to the former rather than to the African *Entypesa* Simon, 1902 as suggested by Raven and Schwendinger (1995).

To permit reliable identification and stimulate further studies in this area, all new descriptions are illustrated and keys to the studied genera and species are added.

## Material and methods

Specimens were examined and measured with a LEICA M205 C stereomicroscope and details were studied with an Olympus BX51 compound microscope. Illustrations were made using a camera lucida attached to the Olympus BX51 microscope and inked with an ink jet plotter. Male palps and female genitalia were examined and illustrated after they were dissected from the spiders. Vulvae were treated in a warm solution of 10% potassium hydroxide (KOH). The left palp and left legs I and II of male spiders were illustrated, unless otherwise indicated. Specimens were preserved in a 75% ethanol solution. Photos were taken with an Olympus C7070 wide zoom digital camera (7.1 megapixels) mounted on an Olympus SZX12 stereomicroscope. The images were assembled using Helicon image stacking software. All measurements are given in millimetres unless otherwise noted. Leg measurements are given as: total length (femur + patella + tibia + metatarsus + tarsus). Leg segments were measured on the dorsal side.

The following abbreviations are used: AL – abdomen length; ALE – anterior lateral eye(s); AME – anterior median eye(s); AW – abdomen width; CL – carapace length; CW – carapace width; PLE – posterior lateral eye(s); PLS – posterior lateral spinneret(s); PME – posterior median eye(s); PMS – posterior median spinneret(s); TL – total length (including chelicerae, but not spinnerets).

All specimens used in this study are deposited in the Institute of Zoology, Chinese Academy of Sciences (IZCAS) in Beijing, China. The only exception is the female paratype of *Raveniola chayi* sp. n., kept at the Senckenberg Museum, Frankfurt am Main, Germany (SMF).

## Taxonomy

### Family Nemesiidae

Taxon classificationAnimaliaAraneae

Simon, 1889

#### Notes.

Only two genera of the family occur with some degree of certainty in eastern Asia. Judging from the original description, *Nemesia sinensis* Pocock, 1901 probably belongs to the Cyrtaucheniidae (see Zonstein and Marusik 2012).

#### Key to the East and South-East Asian genera of Nemesiidae:

The distribution of *Atmetochilus* is given considering data provided by Schwendinger (1996) and Zonstein and Marusik (in press).

**Table:** 

1	Thoracic fovea short, U-shaped; posterior sternal sigilla distinctly larger and farther from sternal margin than other sigilla, in many cases subcentral or confluent; paired tarsal claws either with two more or less distinctly juxtaposed teeth rows (females) or with one S-shaped row (males); PMS well developed; male tibia I with coupling spur and megaspines located proventrally	**2**
–	Thoracic fovea short, straight or pit-like; posterior sternal sigilla submarginal; paired tarsal claws with two similar teeth rows on promargin and retromargin in males and females; PMS small to absent; male tibia I with two enlarged retroventral distal spines and without coupling spur	**4**
2	Posterior sternal sigilla submarginal to subcentral but not confluent (Raven 1985: fig. 53; Siliwal et al. 2015: figs 1D, 2D)	**3**
–	Posterior sternal sigilla confluent; (Raven 1985: fig. 59)	***Atmetochilus*** (India, Indonesia, Myanmar and Thailand)
3	Metatarsal preening combs absent on legs III and IV; female tarsus IV with scopula	***Damarchilus*** (Eastern India)
–	Metatarsal preening combs present on legs III and IV; female tarsus IV without scopula	***Damarchus*** (Eastern India, Indonesia, Malaysia, Myanmar, Singapore and Thailand)
3	Carapace hirsute and with finely granular texture; hairs on legs I–IV long and non-uniform; tarsal scopula more or less dense and long; male intercheliceral tumescence reduced if present	***Raveniola*** (south Palearctic, from Turkey to China)
–	Carapace with only a few bristles; hairs on legs I–IV more or less uniformly short; tarsal scopula thin and short; male intercheliceral tumescence well developed	***Sinopesa*** (South-eastern China, Ryukyu Isles, Thailand and Vietnam)

### Genus Raveniola

Taxon classificationAnimaliaAraneaeNemesiidae

Zonstein, 1987

#### Type species.

*Brachythele virgata* Simon, 1891, from Central Asia, by the original designation.

#### Diagnosis.

*Raveniola*, similar to *Sinopesa* Raven & Schwendinger, 1995, has two enlarged retroventral distal spines on tibia I in males and divided receptacles in females, as well as the absence of a serrula and metatarsal preening combs. The leg scopula in *Raveniola* is more developed than it is in *Sinopesa*. By contrast, the male intercheliceral tumescence in *Raveniola* is lost or vestigial, whereas in *Sinopesa*, it is well-developed. Like *Sinopesa*, members of *Raveniola* have more or less reduced PMS, which are completely lost in some species. The apical segment of the PLS in *Raveniola* is usually shorter than that in *Sinopesa*.

#### Composition.

*Raveniola* currently comprises 29 species, including the new species described here; 14 of them occur in China: *Raveniola alpina* sp. n., *Raveniola bellula* sp. n., *Raveniola chayi* sp. n., *Raveniola gracilis* sp. n., *Raveniola guangxi* (Raven & Schwendinger, 1995), *Raveniola hebeinica* Zhu, Zhang & Zhang, 1999, *Raveniola montana* Zonstein & Marusik, 2012, *Raveniola rugosa* sp. n., *Raveniola shangrila* Zonstein & Marusik, 2012, *Raveniola songi* Zonstein & Marusik, 2012, *Raveniola spirula* sp. n., *Raveniola xizangensis* (Hu & Li, 1987), *Raveniola yajiangensis* sp. n. and *Raveniola yunnanensis* Zonstein & Marusik, 2012.

#### Key to East Asian Raveniola species

Females of *Raveniola alpina* sp. n., *Raveniola gracilis* sp. n., *Raveniola guangxi*, *Raveniola rugosa* sp. n., *Raveniola shangrila*, *Raveniola songi*, *Raveniola spirula* sp. n. and *Raveniola yunnanensis* are unknown.

**Table:** 

1	Males	**2**
–	Females	**15**
2	PMS present	**3**
–	PMS absent	**9**
3	Carapace length > 10 mm. Embolus with distinct subapical keel	***xizangensis***
–	Carapace length 3.0–7.3 mm. Embolic keel absent or vestigial	**4**
4	Palpal tibia relatively short, with a length/width ratio of 3.0–3.2 (Fig. 11A–C). Embolus short and with deep subbasal ridges (Fig. 12A–C)	***montana***
–	Palpal tibia and embolus relatively long, with a length/width ratio of 3.6–4.5 (Figs 1A–C, 2A–C, 3A–C, 5A–C, 6A–C, 7A–C, 9A–C, 10A–C, 13A–C, 14A–C, 15A–C, 16A–C, 17A–C, 19A–C)	**5**
5	Embolus with distally hooked tip (Figs 9A–C, 10A–C)	***gracilis* sp. n.**
–	Embolic tip not hooked	**6**
6	Embolus more or less twisted	**7**
–	Distal part of embolus curved gradually (Zonstein and Marusik 2012: fig. 39)	***hebeinica***
7	Tibia I equal in length to or shorter than metatarsus (as in Fig. 17E). Few spines on cymbium (as in Fig. 19A–C)	**8**
–	Tibia I considerably longer than metatarsus (see Zonstein and Marusik 2012: fig. 29). Cymbium with numerous dorsal spines (Op. cit.: fig. 35)	***yunnanensis***
8	Embolus only slightly twisted (Op. cit.: fig. 42)	***songi***
–	Embolus distinctly twisted (Figs 19A–C)	***yajiangensis* sp. n.**
9	Embolus with hooked tip (see Zonstein and Marusik 2012: figs 37, 38)	***guangxi***
–	Embolic tip not hooked	**10**
10	Embolus strongly spiralled as in Figs 15A–C, 16A–C	***spirula* sp. n.**
–	Embolus more or less curved, slightly spiralled or bent as in Figs 1A–C, 2A–C, 3A–C, 5A–C, 6A–C, 7A–C, 13A–C, 14A–C	**11**
11	Embolus slightly spiralled as in Figs 1A–C, 2A–C	***alpina* sp. n.**
–	Embolus curved or gradually twisted	**12**
12	Entire embolus arched as in Figs 13A–C, 14A–C	***rugosa* sp. n.**
–	Embolus more or less distinctly twisted as in Figs 3A–C, 5A–C, 6A–C, 7A–C	**13**
13	Abdomen with dorsal and ventral spotted pattern (Figs 3D, G, 6D, G). Embolus only slightly twisted (Figs 3A–C, 5A–C, 6A–C, 7A–C)	**14**
–	Abdomen uniformly dark brown. Embolus noticeably twisted (see Zonstein and Marusik 2012: fig. 41)	***shangrila***
14	Dorsal abdominal pattern consists of numerous darker spots on a lighter background (Fig. 3D). Metatarsus I very gently curved (Fig. 3E). Embolus moderately long without ridges (Figs 3A–C, 5A–C)	***bellula* sp. n.**
–	Dorsal abdominal pattern consists of numerous lighter spots on a darker background (Fig. 6D). Metatarsus I noticeably curved (Fig. 6E). Embolus long and tapering with ridges (Figs 6A–C, 7A–C)	***chayi* sp. n.**
15	PMS present	**16**
–	PMS absent	**19**
16	Carapace length > 10 mm. Median (ental) branch of receptacle bifurcate (Op. cit., fig. 50)	***xizangensis***
–	Carapace length < 8 mm. Median (ental) branch of receptacle entire	**17**
17	Median (ental) branch of receptacle curved inward as shown in Figs 18, 19D	***yajiangensis* sp. n.**
–	Shape of receptacles different	**18**
18	PLS: apical segment triangular. Receptacular bases narrow (Zonstein and Marusik 2012, fig. 47)	***hebeinica***
–	PLS: apical segment digitiform. Receptacular bases widened (Op. cit., fig. 48)	***montana***
19	Receptacles as shown in Figs 4A, 5D	***bellula* sp. n.**
–	Receptacles as shown in Figs 8B–C	***chayi* sp. n.**

### Raveniola alpina sp. n.

Taxon classificationAnimaliaAraneaeNemesiidae

http://zoobank.org/E841EDAB-BDA2-49FC-B8B8-9C435C8303B3

Figs 1 , 2

#### Type material.

Holotype ♂ – CHINA, Yunnan Province, Zhongdian County, northern Zhongdian [27°50.119'N, 99°42.426'E, elevation 3285 m], July 23–30, 2000, X. Yu & H. Zhou (IZCAS). Paratypes: same data but Xiaoxueshanyakou [27°49.119'N, 99°41.426'E, elevation 3265 m] – 1♂ (IZCAS); same data but Bitahaixi [27°48.105'N, 99°40.429'E, elevation 3285 m] – 2♂ (IZCAS).

#### Etymology.

The specific name is taken from the Latin adjective “*alpinus*”, which means “alpine” and refers to the high altitude of the type locality.

#### Diagnosis.

**Figure 1.**
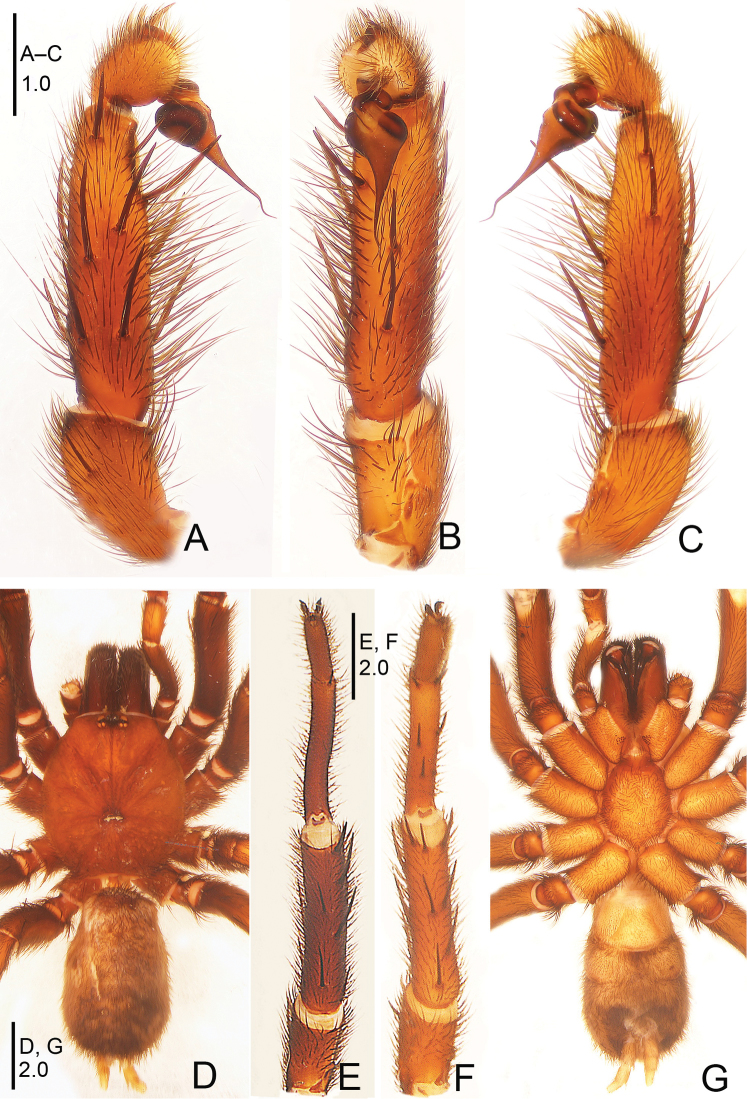
*Raveniola alpina* sp. n., male holotype. **A** palp, prolateral view **B** palp, ventral view **C** palp, retrolateral view **D** habitus, dorsal view **E** leg I, ventral view **F** leg II, ventral view **G** habitus, ventral view. Scale bars: 1.0 mm (**A–C**); 2.0 mm (**D–G**).

**Figure 2.**
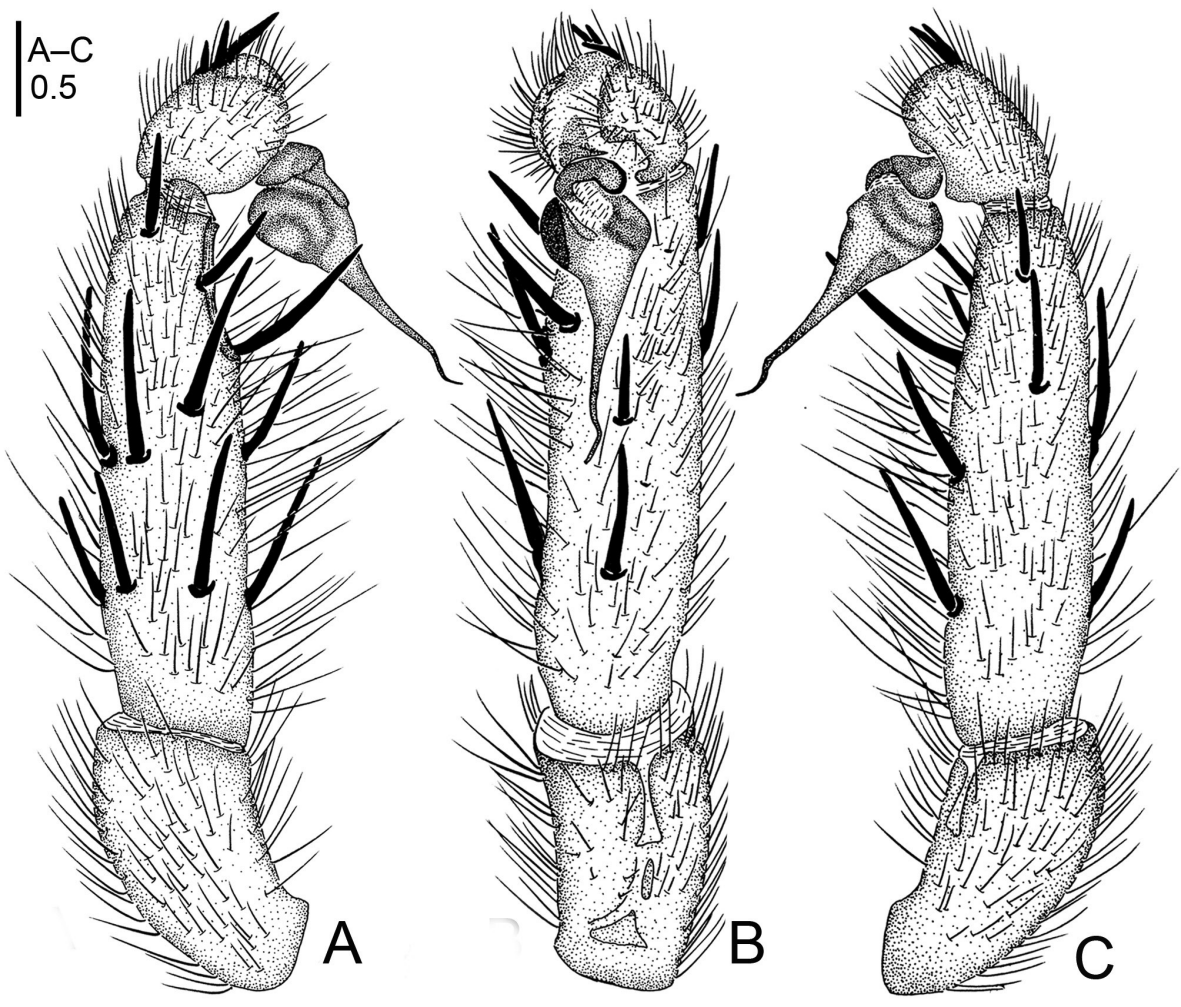
*Raveniola alpina* sp. n., male holotype. **A** palp, prolateral view **B** palp, ventral view **C** palp, retrolateral view. Scale bar: 0.5 mm.

The new species is similar to *Raveniola chayi* sp. n., *Raveniola shangrila* and *Raveniola songi*, all also occurring in Yunnan, but differs by the slightly twisted and bent distal portion of the embolus (Figs 1A–C, 2A–C, *cf.* Figs 6A–C, 7A–C). *Raveniola alpina* sp. n. can be distinguished from the latter species also by the absence of PMS (present in *Raveniola songi*).

#### Description.

Male (holotype): TL 14.50, CL 5.75, CW 5.25, AL 6.55, AW 4.00. Eye diameters and interdistances: AME 0.17, ALE 0.31, PME 0.13, PLE 0.15, AME–AME 0.19, AME–ALE 0.10, PME–PME 0.55, PME–PLE 0.08. Leg lengths: I: 17.05 (4.90+2.10+4.40+3.50+2.15), II: 16.15 (4.85+1.50+4.30+3.40+2.10), III: 14.50 (4.25+1.70+3.10+3.25+2.20), IV: 18.45 (5.10+2.15+4.55+4.10+2.55). Labium, sternum and maxillae as shown in Fig. 1G. Maxillae with 15–20 cuspules. Prosoma, palps and legs brown. Spinnerets deep grey (Fig. 1D, G). Metatarsus I noticeably curved (Fig. 1E). PMS entirely reduced, apical segment of PLS digitiform (Fig. 1D, G). Palpal tibia long, cylindrical; bulb long, pyriform; embolus gradually tapering to a slender bent point; distal cymbium with three short, stout spines (Figs 1A–C, 2A–C).

Female. Unknown.

#### Distribution.

China: northern Yunnan.

### Raveniola bellula sp. n.

Taxon classificationAnimaliaAraneaeNemesiidae

http://zoobank.org/CAB030BD-41D7-43E9-978F-0C0FE340CE19

Figs 3 , 4 , 5

#### Type material.

Holotype ♂ – CHINA, Yunnan Province, Mengla County, Xishuangbanna, Menglun Town, primary tropical seasonal rainforest in Menglun Nature Reserve [21°57.445'N, 101°12.997'E, 744 m], January 16–31, 2007, G. Zheng (IZCAS). Paratypes: 26♂, 2♀ (IZCAS), same data as holotype.

#### Etymology.

The specific name is taken from the Latin adjective “bellulus” (the diminutive form of “bellus”), which means “beautiful” and refers to the perfect shape of the palpal bulb.

#### Diagnosis.

**Figure 3.**
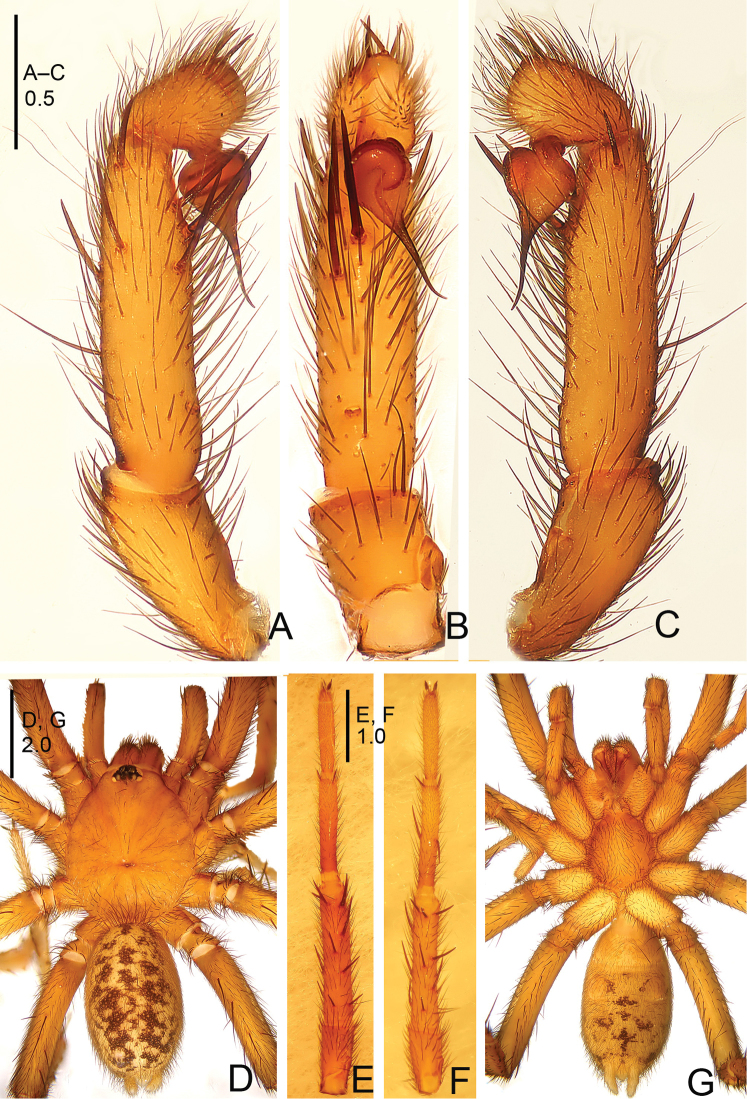
*Raveniola bellula* sp. n., male holotype. **A** palp, prolateral view **B** palp, ventral view **C** palp, retrolateral view **D** habitus, dorsal view **E** leg I, ventral view **F** leg II, ventral view **G** habitus, ventral view. Scale bars: 0.5 mm (**A–C**); 2.0 mm (**D, G**); 1.0 mm (**E, F**).

**Figure 4.**
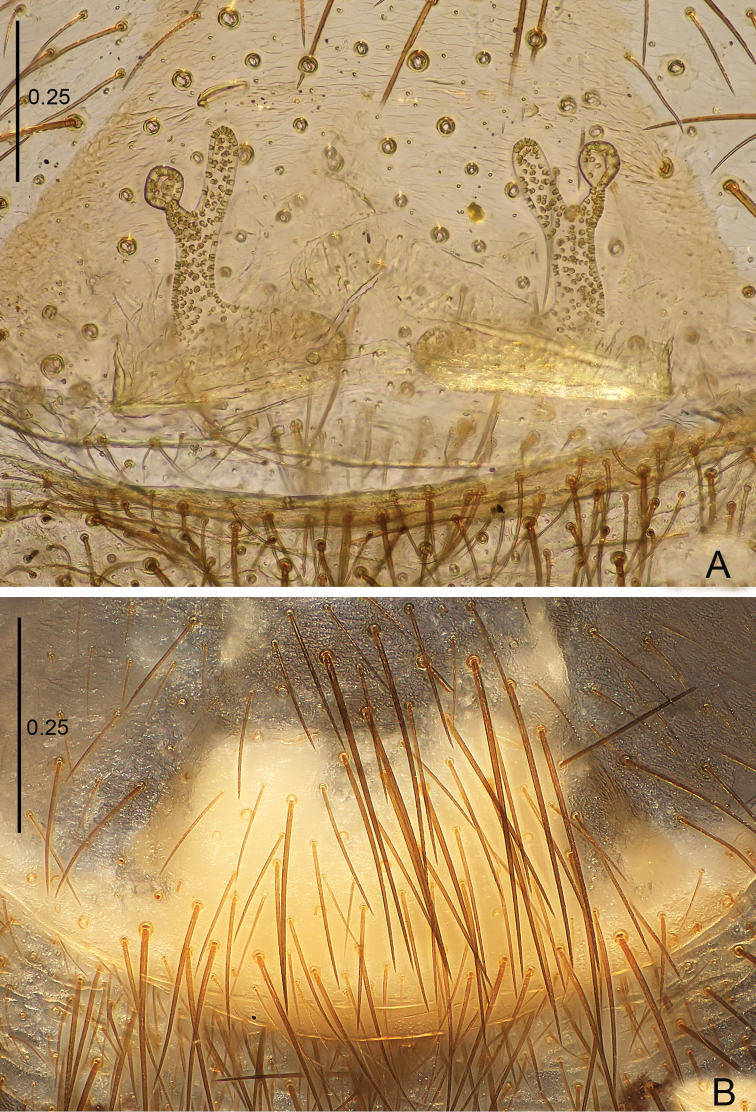
*Raveniola bellula* sp. n., female paratype. **A** vulva, dorsal view **B** genital area, ventral view. Scale bars: 0.25 mm.

**Figure 5.**
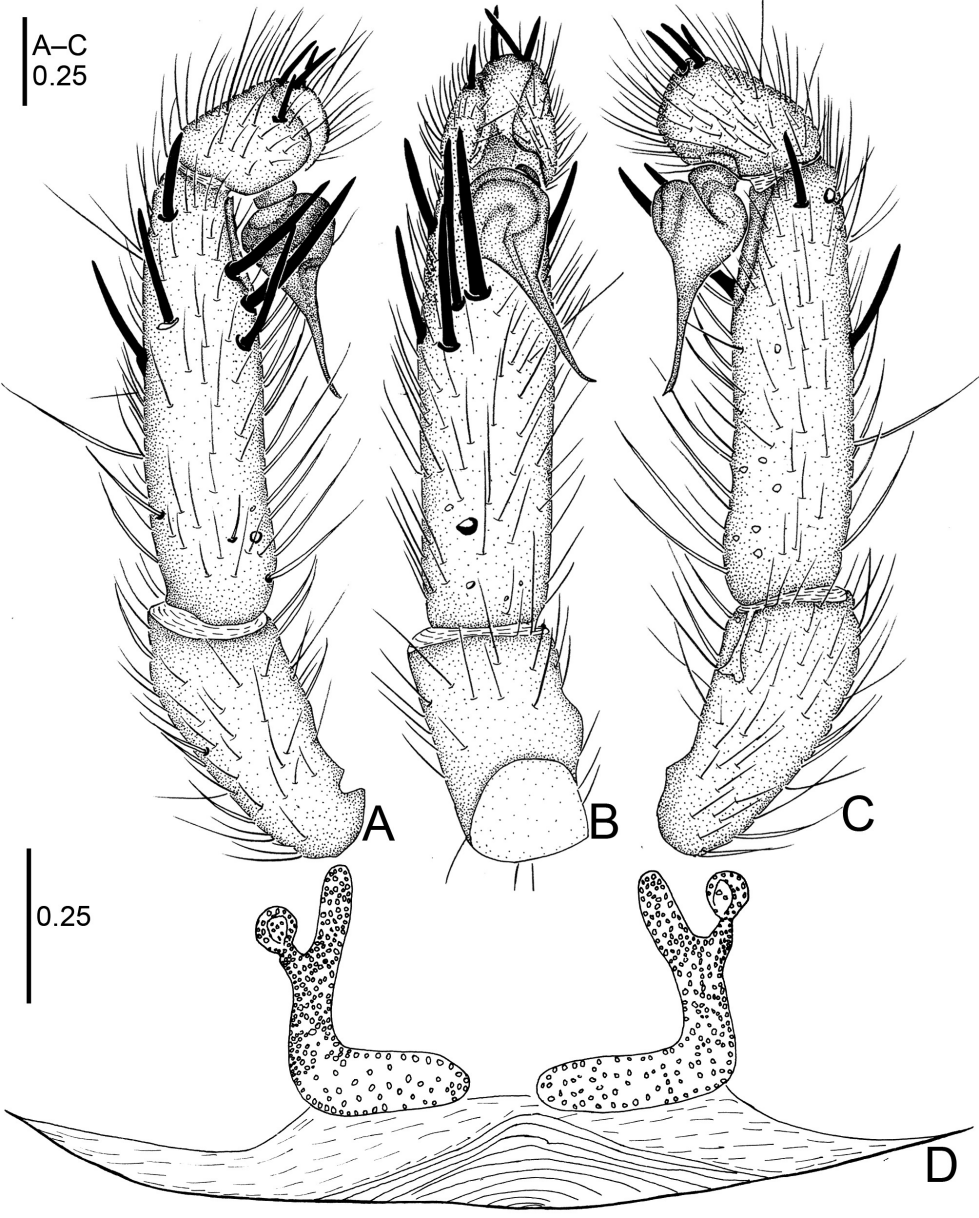
*Raveniola bellula* sp. n., male holotype (**A–C**) and female paratype (**D**). **A** palp, prolateral view **B** palp, ventral view **C** palp, retrolateral view **D** vulva, dorsal view. Scale bars: 0.25 mm.

**Figure 6.**
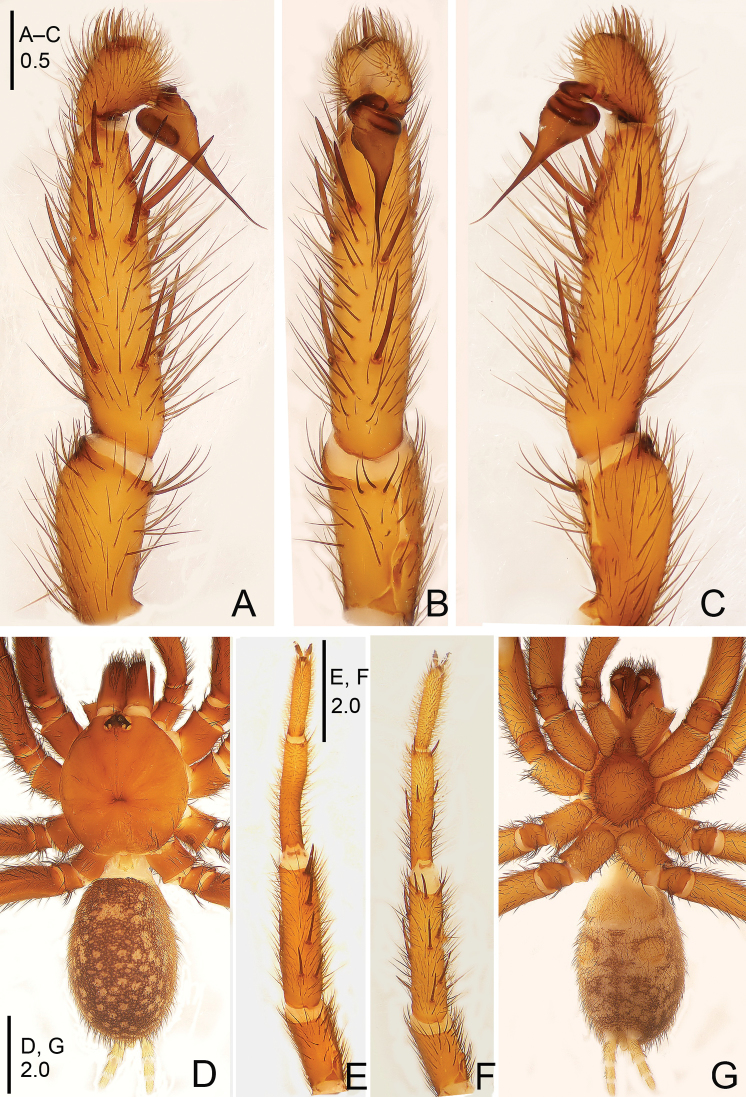
*Raveniola chayi* sp. n., male holotype. **A** palp, prolateral view **B** palp, ventral view **C** palp, retrolateral view **D** habitus, dorsal view **E** leg I, ventral view **F** leg II, ventral view **G** habitus, ventral view. Scale bars: 0.5 mm (**A–C**); 2.0 mm (**D–G**).

**Figure 7.**
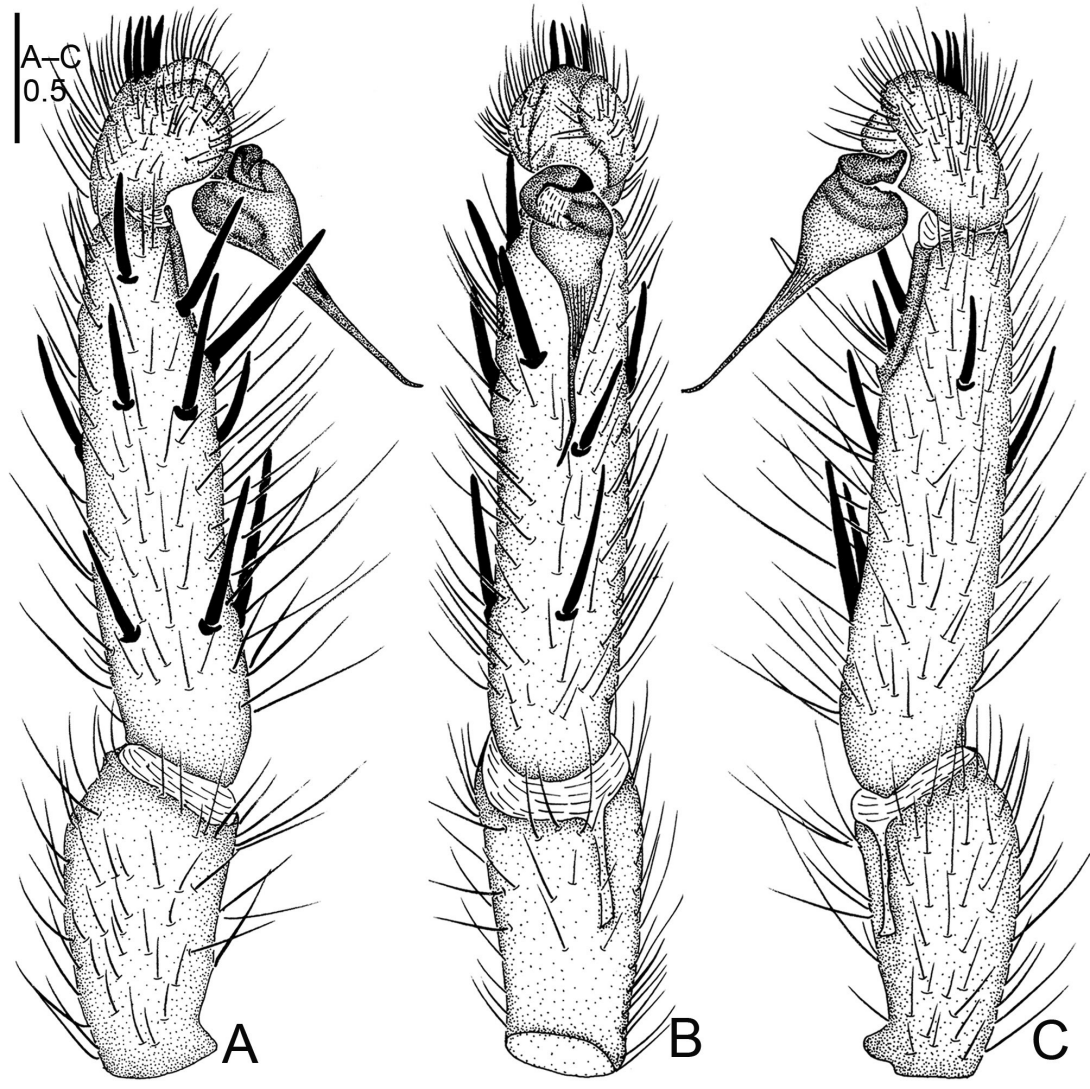
*Raveniola chayi* sp. n., male holotype. **A** palp, prolateral view **B** palp, ventral view **C** palp, retrolateral view. Scale bar: 0.5 mm.

This new species is similar to *Raveniola chayi* sp. n. and *Raveniola yunnanensis* but can be distinguished by having a considerably shorter embolus than that in *Raveniola chayi* sp. n. (Figs 3A–C, 5A–C; *cf.* Figs 6A–C, 7A–C), by possessing a longer cymbium and a less twisted embolus than *Raveniola yunnanensis*, as well as by having a ventral abdominal pattern and completely reduced PLS (Fig. 3A–C; *cf.* Zonstein and Marusik 2012: figs 35, 43). Females are characterised by the unique shape of the receptacles, divided into a long, digitiform inner branch and a short, knob-shaped outer branch (Figs 4A, 5D). The habitus and the abdominal pattern of *Raveniola bellula* sp. n. resemble that of *Sinopesa maculata*, but it is distinguished by generic characters, such as well-developed body and leg setation and by much longer and denser tarsal scopula.

#### Description.

Male (holotype): TL 7.90, CL 3.50, CW 2.55, AL 3.60, AW 2.25. Eye diameters and interdistances: AME 0.20, ALE 0.24, PLE 0.16, PME 0.12, AME–AME 0.09, AME–ALE 0.04, PME–PME 0.35, PME–PLE 0.03. Leg lengths: I: 10.04 (2.75+1.65+2.65+1.80+1.55), II: 9.70 (2.75+1.30+2.30+1.85+1.50), III: 9.05 (2.50+1.15+1.65+2.25+1.50), IV: 12.40 (3.25+1.25+3.10+3.15+1.65). Labium, sternum and maxillae as shown in Fig. 3G. Maxillae with *ca.* 20 cuspules. Prosoma, palps and legs light brown. Spinnerets light grey. Light yellowish grey abdomen with darker (brown) dorsal and ventral pattern (Fig. 3D, G). Metatarsus I very slightly curved (Fig. 3E). PMS absent; apical segment of PLS digitiform (Fig. 3D, G). Palpal tibia moderately long, subcylindrical; bulb long, oval; embolus gradually tapering to a slender point; cymbium with four short, stout spines (Figs 3A–C, 5A–C).

Female. TL 6.75, CL 3.25, CW 2.50, AL 2.75, AW 2.40; body and legs colouration as in male. Eye diameters and interdistances: AME 0.22, ALE 0.24, PME 0.10, PLE 0.15, AME–AME 0.08, AME–ALE 0.05, PME–PME 0.36, PME–PLE 0.04, Leg lengths: I: 10.15 (2.65+1.60+2.55+1.90+1.45), II: 9.55 (2.65+1.45+2.25+1.75+1.45), III: 8.75 (2.45+1.10+1.50+2.25+1.45), IV: 12.00 (3.20+1.15+3.00+3.10+1.55). Genital area as in Fig. 4B. Receptacles divided into a long, digitiform inner branch and a short, knob-shaped outer branch that is bent forward (Figs 4A, 5D).

#### Variation.

Total length of males and females: 6.75–7.93 (n=8).

#### Distribution.

The species is known only from the type locality.

### Raveniola chayi sp. n.

Taxon classificationAnimaliaAraneaeNemesiidae

http://zoobank.org/DE55EA80-52FE-464E-B37E-4D25F7C38DA9

Figs 6 , 7 , 8

#### Type material.

Holotype ♂ – CHINA: Yunnan Province, Lijiang County, Mt. Yulongxueshan, Maoniuping [27°05.503'N, 100°15.403'E, elevation 3061 m], August 1–3, 2000, X. Yu (IZCAS). Paratypes: 16♂ (IZCAS), same data as holotype; 1♀ – Sichuan Province, Yanyuan County, around Lugu Lake [27°48'N, 100°49'E, elevation 3300 m], May 28, 2011, J. Martens (SMF).

#### Etymology.

The specific name is from the Chinese word for difference (chā yì), in reference to the difference between the new species with *Raveniola songi* and *Raveniola yunnanensis*; noun.

#### Diagnosis.

The new species is similar to *Raveniola alpina* sp. n., *Raveniola songi* and *Raveniola yunnanensis* but can be distinguished by the smooth distal portion of the embolus (Figs 6A–C, 7A–C), the presence of 4 spines on the cymbium (Figs 6A–C, 7A–C) and the presence of ridges on the proximal portion of the embolus (Figs 6A–C, 7A–C); it can be distinguished from latter two congeners by lacking the PMS (present in those species).

#### Description.

Male (holotype): TL 10.30, CL 4.30, CW 3.65, AL 4.90, AW 3.10. Eye diameters and interdistances: AME 0.17, ALE 0.23, PME 0.13, PLE 0.21; AME–AME 0.11, AME–ALE 0.06, PME–PME 0.31, PME–PLE 0.05. Leg lengths: I: 12.50 (3.60+1.25+2.95+2.45+2.25), II: 11.65 (3.55+1.25+2.85+2.50+1.50), III: 10.55 (2.55+1.10+2.50+2.65+1.75), IV: 14.40 (4.00+1.30+3.10+4.05+1.95). Maxillae, labium and sternum as shown in Fig. 6G. Maxillae with 12–15 cuspules. Prosoma, palps and legs light brown. Spinnerets light grey, abdomen brown with light dorsal and ventral spots (Fig. 6D, G). Metatarsus I noticeably curved (Fig. 6E). PMS absent; apical segment of PLS digitiform (Fig. 6D, G). Palpal tibia long, subcylindrical; cymbium with four short, stout spines; bulb long, pyriform; embolus gradually tapering to a slender point (Figs 6A–C, 7A–C).

**Figure 8.**
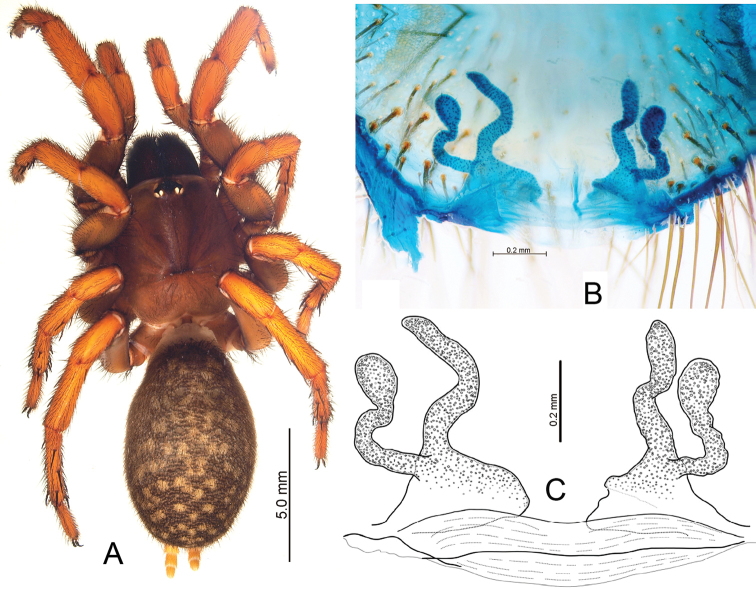
*Raveniola chayi* sp. n., female paratype. **A** habitus, dorsal view **B, C** vulva, dorsal view. Scale bars: 5.0 mm (**A**); 0.2 mm (**B–C**).

Female (paratype): TL 14.75, CL 5.75, CW 4.90, AL 9.00, AW 5.25. Eye diameters and interdistances: AME 0.17, ALE 0.30, PME 0.17, PLE 0.23, AME–AME 0.21, AME–ALE 0.12, PME–PME 0.47, PME–PLE 0.04. Leg lengths: I: 13.15 (4.20+2.25+3.00+2.30+1.40), II: 11.95 (3.70+2.20+2.35+2.30+1.40), III: 11.90 (3.15+2.00+2.05+3.05+1.65), IV: 15.90 (4.25+2.30+3.10+4.30+1.95). Most characters, including the colouration peculiarities (Fig. 8A) and the absence of PMS, are as in the male. Receptacles divided into a stocking-shaped inner branch and a clubbed outer branch; both branches long and crimped (Fig. 8B–C).

#### Variation.

Total length of males: 9.49–11.10 (n=8).

#### Distribution.

China: north-western Yunnan, south-western Sichuan.

### Raveniola gracilis sp. n.

Taxon classificationAnimaliaAraneaeNemesiidae

http://zoobank.org/1CC980B2-6464-44C3-9225-F5AD0BBE168C

Figs 9 , 10

#### Type material.

Holotype ♂ – CHINA: Zhejiang Province, Hangzhou County, Hangzhou [30°16.276'N, 120°09.178'E, 260 m], July 1980, Z. Chen (IZCAS).

#### Etymology.

The specific name is taken from the Latin adjective “*gracilis*”, which means “slender” and refers to the shape of embolus.

#### Diagnosis.

**Figure 9.**
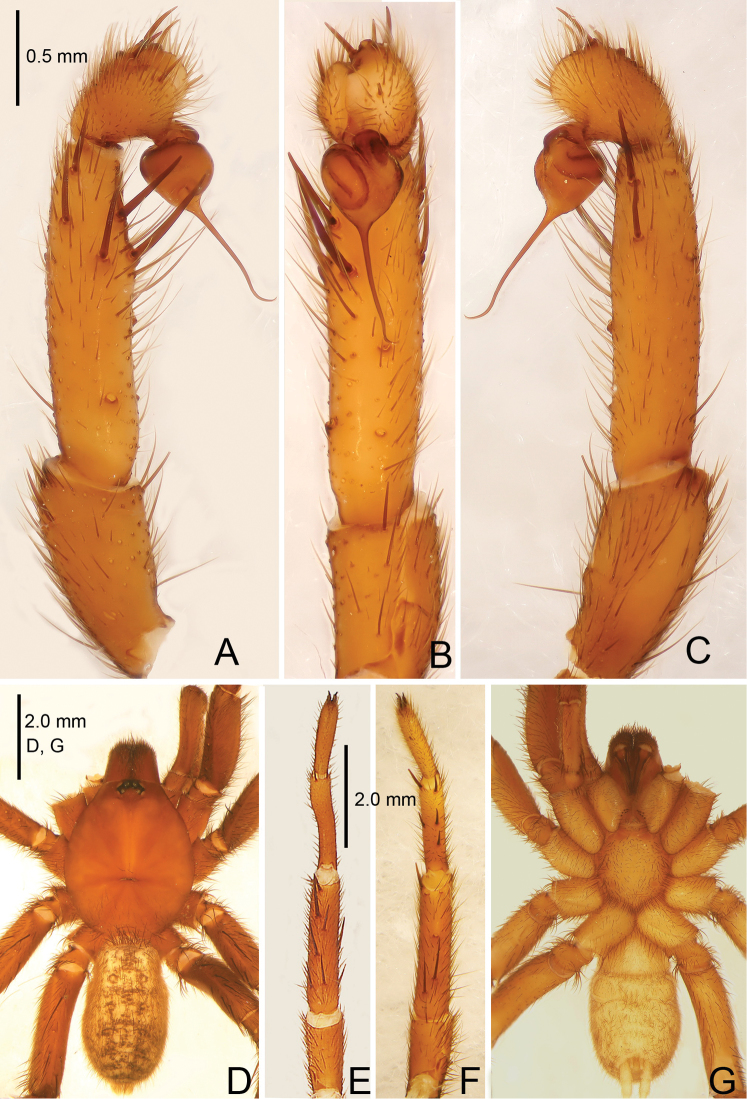
*Raveniola gracilis* sp. n., male holotype. **A** palp, prolateral view **B** palp, ventral view **C** palp, retrolateral view **D** habitus, dorsal view **E** leg I (right side), ventral view **F** leg II, ventral view **G** habitus, ventral view. Scale bars: 0.5 mm (**A–C**); 2.0 mm (**D–G**).

**Figure 10.**
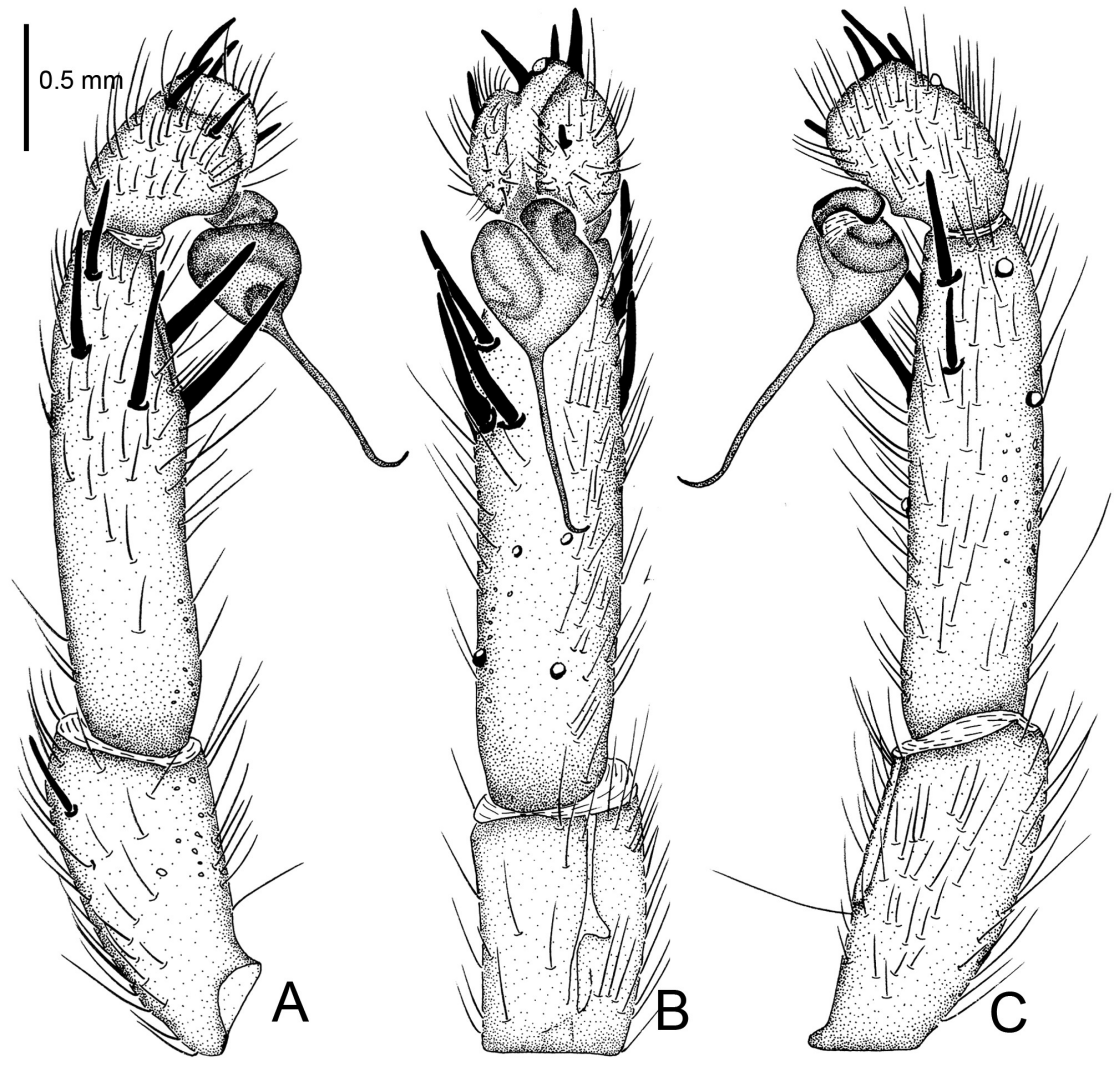
*Raveniola gracilis* sp. n., male holotype. **A** palp, prolateral view **B** palp, ventral view **C** palp, retrolateral view. Scale bar: 0.5 mm.

This new species can be easily distinguished from all known congeners by its slender and subapically curved embolus (Figs 9A–C, 10A–C).

#### Description.

Male (holotype): TL 8.30, CL 4.05, CW 3.25, AL 3.90, AW 2.40. Eye sizes and interdistances: AME 0.15, ALE 0.22, PME 0.11, PLE 0.15, AME–AME 0.11, AME–ALE 0.07, PME–PME 0.34, PME–PLE 0.04. Leg lengths: I: 11.90 (3.50+1.55+2.95+2.15+1.75), II: 11.00 (3.05+1.60+2.70+1.80+1.85), III: 10.75 (2.75+1.60+2.25+2.25+1.60), IV: 12.85 (3.25+1.60+3.15+3.10+1.75). Carapace yellowish brown dorsally, with a few brownish setae. Eye tubercle blackish brown. Chelicerae reddish dark brown. Sternum, labium, maxillae and legs light brown ventrally. Abdomen dorsally light brown, with blackish cloudy maculae and brownish setae. Ventral surface of abdomen and spinnerets yellowish brown, with dense brownish setae (Fig. 9D, G). Fovea broad, slightly recurved (Fig. 9D). Chelicerae without rastellum but with strong setae (Fig. 9D, G). Maxillae with 7–9 cuspules. Three pairs of cloudy sternal sigilla (Fig. 9G). Leg tarsi without spines. Tarsal claws with two rows of uniform teeth. Metatarsus I curved and bent (Fig. 9E). Two pairs of spinnerets, apical segment of PLS digitiform. Tip of cymbium with 5 strong spines. Bulb smooth, pyriform, with long, slender embolus (Figs 9A–C, 10A–C).

Female. Unknown.

#### Distribution.

The species is known only from the type locality.

### Raveniola montana

Taxon classificationAnimaliaAraneaeNemesiidae

Zonstein & Marusik, 2012

Figs 11 , 12

#### Material.

CHINA: Sichuan Province, Baoxing County, Baoxing [30°22.052'N, 102°48.534'E, elevation 1115 m], June 2001, X. Yu & H. Zhou – 3♂ (IZCAS); same county, Qiaoqi Town [30°41.129'N, 102°42.370'E, 2447 m], June 6–7, 1997, leg. Z. Zhang.

#### Diagnosis.

**Figure 11.**
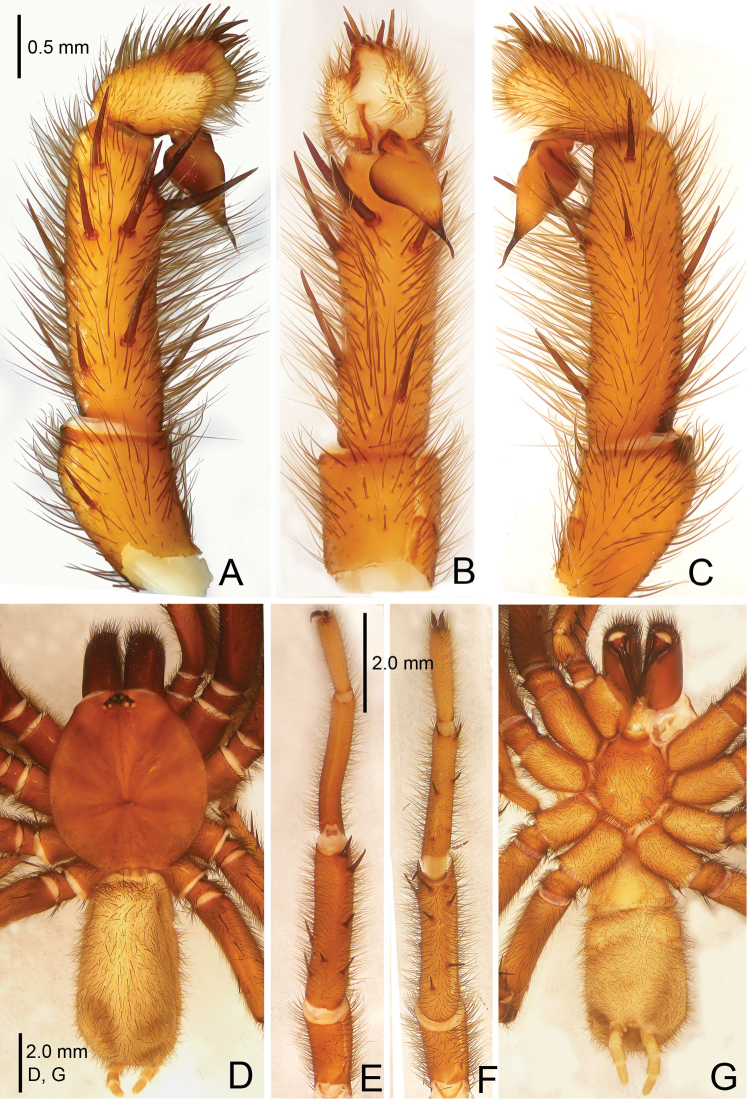
*Raveniola montana* Zonstein & Marusik, 2012, male (Sichuan Prov.). **A** palp, prolateral view **B** palp, ventral view **C** palp, retrolateral view **D** habitus, dorsal view **E** leg I, ventral view **F** leg II, ventral view **G** habitus, ventral view. Scale bars: 0.5 mm (**A–C**); 2.0 mm (**D–G**).

**Figure 12.**
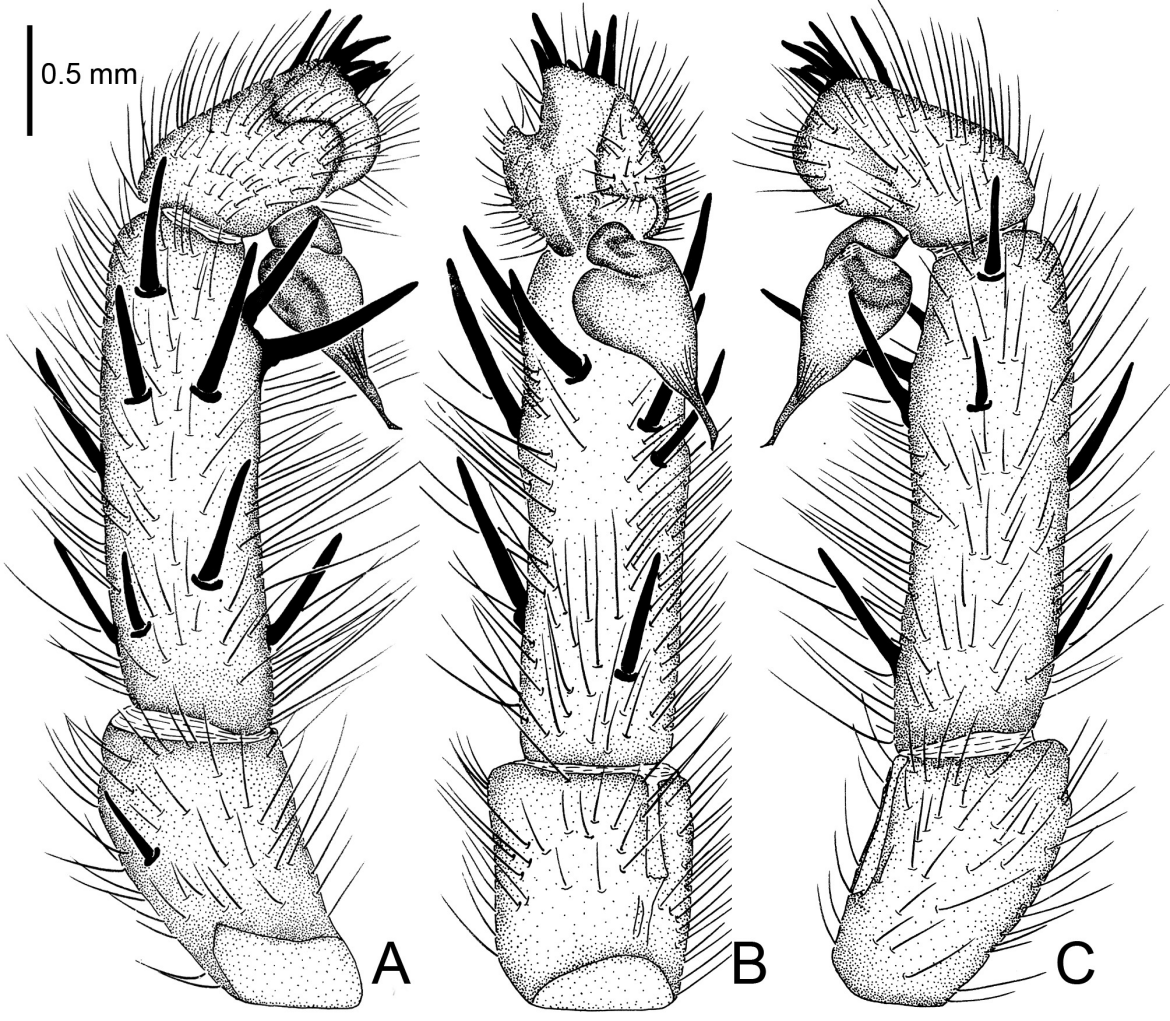
*Raveniola montana* Zonstein & Marusik, 2012, male (Sichuan Prov.). **A** palp, prolateral view **B** palp, ventral view **C** palp, retrolateral view. Scale bar: 0.5 mm.

This species can be easily distinguished from all known East Asian congeners by its short and stout palpal tibia and by a short and flattened embolus (Fig. 11A–C), combined with the presence of ridges on the wide proximal portion of the embolus (Fig. 12A–C) and the unique shape of the receptacles in females (Zonstein and Marusik 2012: fig. 48).

#### Description.

See Zonstein and Marusik (2012): 77, figs 5, 9, 10, 17, 18, 26, 32, 40, 48.

#### Variation.

Total length: 10.46–11.56 in males from Sichuan (n=8) *vs.* 15.50 in the male holotype from Yunnan.

#### Distribution.

China: northern Yunnan and southern Sichuan.

### Raveniola rugosa sp. n.

Taxon classificationAnimaliaAraneaeNemesiidae

http://zoobank.org/307964FF-DEC2-410A-9CDF-8CA41D5D924E

Figs 13 , 14

#### Type material.

Holotype ♂ – CHINA: Yunnan Province, Lijiang County, Shigu Town, Shigu east [26°52.014'N, 100°13.588'E, elevation 2393 m], July 31 to August 4, 2007, X. Yu (IZCAS).

#### Etymology.

The specific name is taken from the Latin adjective “*rugosus*”, meaning “wrinkled” and refers to the wrinkled transition between embolus and bulb.

#### Diagnosis.

The new species is similar to *Raveniola chayi* sp. n. but can be distinguished by its embolus gradually curved to the tip (twisted in the latter species) and by the considerably better developed embolic ridges (Figs 7A–C; *cf.* 14A–C). It can be distinguished from *Raveniola montana*, which also possesses embolic ridges on the bulb, by a much longer palpal tibia and a longer embolus (Figs 12A–C; *cf.* 14A–C).

#### Description.

**Figure 13.**
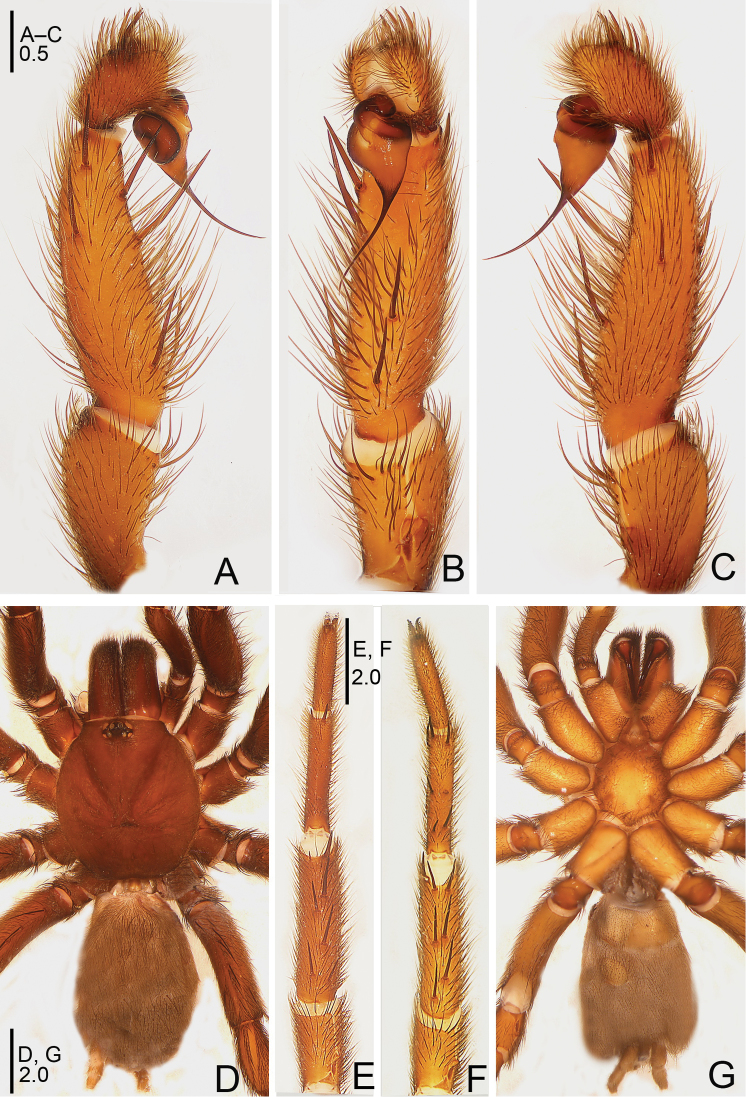
*Raveniola rugosa* sp. n., male holotype. **A** palp, prolateral view **B** palp, ventral view **C** palp, retrolateral view **D** habitus, dorsal view **E** leg I, ventral view **F** leg II, ventral view **G** habitus, ventral view. Scale bars: 0.5 mm (**A–C**); 2.0 mm (**D–G**).

**Figure 14.**
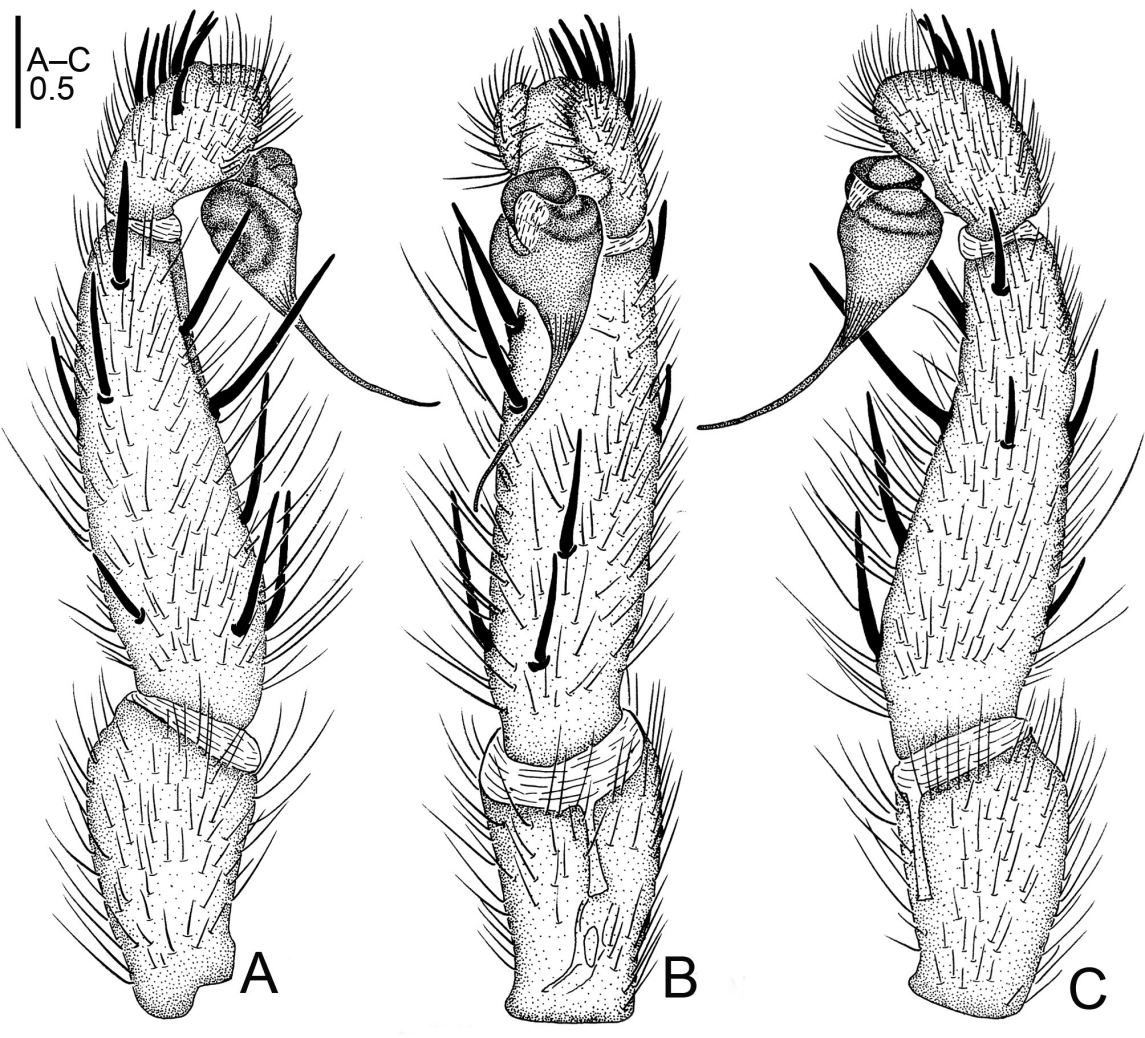
*Raveniola rugosa* sp. n., male holotype. **A** palp, prolateral view **B** palp, ventral view **C** palp, retrolateral view. Scale bar: 0.5 mm.

Male (holotype): TL 14.50, CL 6.15, CW 4.65, AL 6.25, AW 4.00. Eye diameters and interdistances: AME 0.20, ALE 0.24, PME 0.17, PLE 0.19, AME–AME 0.09, AME–ALE 0.04, PME–PME 0.36, PME–PLE 0.06. Leg lengths: I: 16.50 (4.60+2.25+4.25+3.10+2.30), II: 13.95 (4.10+1.60+3.55+2.55+2.15), III: 12.70 (3.75+1.25+2.75+2.70+2.25), IV: 16.95 (4.55+1.60+4.25+4.05+2.50). Venter as shown in Fig. 13G. Maxillae with numerous (*ca.* 35–40) cuspules. Prosoma, palps and legs reddish brown. Abdomen, including spinnerets, light brownish grey (Figs 13D, G). Metatarsus I very gently curved (nearly straight) as in Fig. 13E. PMS absent, apical segment of PLS digitiform (Fig. 13D, G). Palpal tibia slightly swollen at base and slightly arcuate; cymbium with six short, stout spines; bulb long, pyriform; embolus slightly and evenly bent, gradually tapering to a slender point (Figs 13A–C, 14A–C).

Female. Unknown.

#### Distribution.

The species is known only from the type locality.

### Raveniola spirula sp. n.

Taxon classificationAnimaliaAraneaeNemesiidae

http://zoobank.org/C2F3F8EF-C26B-4677-8681-CBB7739959D3

Figs 15 , 16

#### Type material.

Holotype ♂ – CHINA: Hubei Province, Shennongjia Forest Region, Mt. Guanmenshan [31°25.483'N, 110°21.565'E, elevation 1601 m], July 23–30, 1998, H. Zhou (IZCAS). Paratypes: 22♂ (IZCAS), same data as holotype.

#### Etymology.

The specific name is taken from the Latin noun “*spirula*” (the diminutive form of “spira = spiral”), which means “small spiral” and refers to the spiral embolus.

#### Diagnosis.

The new species is similar to *Raveniola yunnanensis* but can be distinguished by a noticeably longer and less spinose cymbium, by its more twisted, corkscrew-shaped distal portion of the embolus (see Fig. 16A–C and Zonstein and Marusik 2012: figs 35, 43) and by the absence of PMS (present in the latter species).

#### Description.

**Figure 15.**
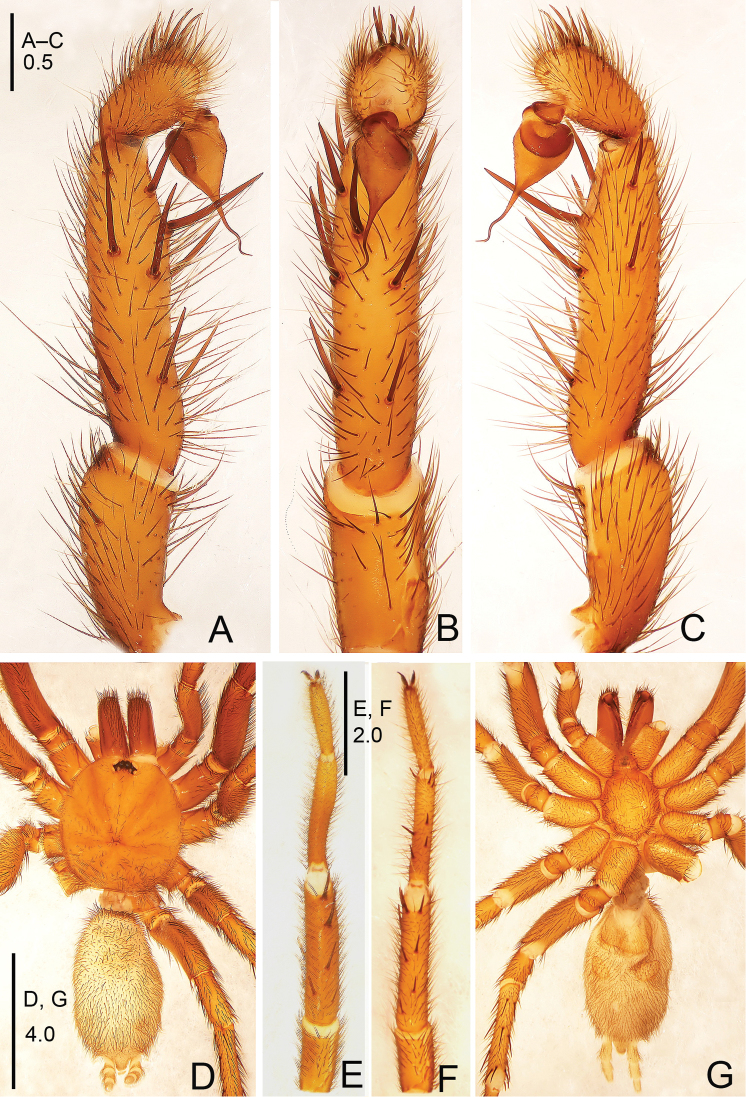
*Raveniola spirula* sp. n., male holotype. **A** palp, prolateral view **B** palp, ventral view **C** palp, retrolateral view **D** habitus, dorsal view **E** leg I, ventral view **F** leg II, ventral view **G** habitus, ventral view. Scale bars: 0.5 mm (**A–C**); 4.0 mm (**D, G**); 2.0 mm (**E, F**).

**Figure 16.**
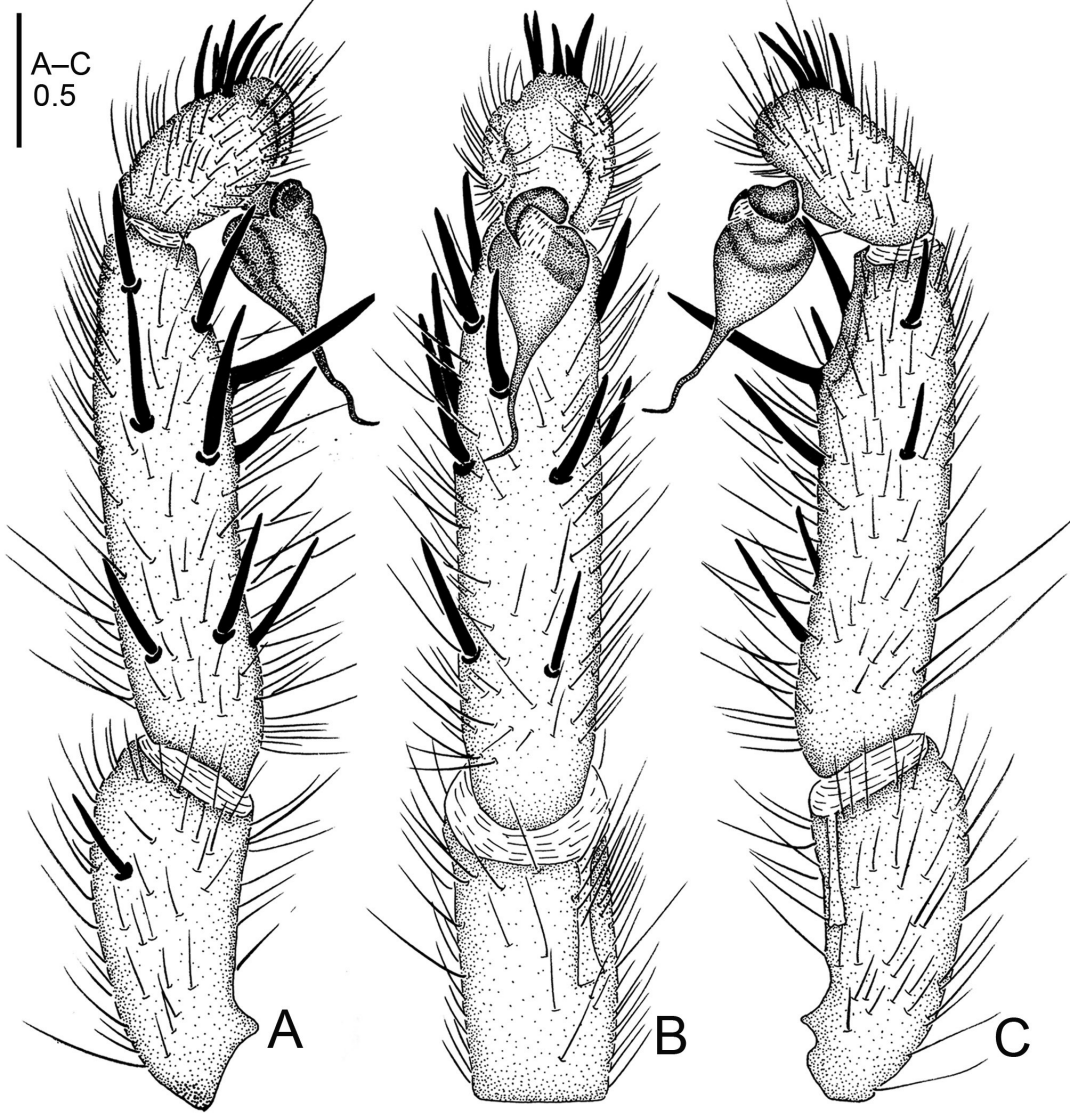
*Raveniola spirula* sp. n., male holotype. **A** palp, prolateral view **B** palp, ventral view **C** palp, retrolateral view. Scale bar: 0.5 mm.

Male (holotype): TL 11.25, CL 3.95, CW 3.50, AL 5.65, AW 2.25. Eye diameters and interdistances: AME 0.11, ALE 0.26, PME 0.09, PLE 0.10, AME–AME 0.08, AME–ALE 0.05, PME–PME 0.28, PME–PLE 0.04. Leg lengths: I: 11.65 (3.25+1.45+3.10+2.30+1.55), II: 10.55 (3.10+1.25+2.55+2.15+1.50), III: 9.65 (2.55+1.05+2.05+2.55+1.75), IV: 13.55 (3.35+1.30+2.75+4.10+2.05). Venter as shown in Fig. 15G. Maxillae with 5 cuspules. Prosoma, palps and legs light brown. Abdomen including spinnerets light grey. Metatarsus I curved outwards (retrolaterally) as in Fig. 15E. PMS absent, apical segment of PLS digitiform (Fig. 15D, G). Palpal tibia long, subcylindrical; cymbium with five short, stout spines; bulb pyriform; embolus strongly twisted, corkscrew-shaped. (Figs 15A–C, 16A–C).

Female. Unknown.

#### Variation.

Total length: 10.46–11.56 (n=8).

#### Distribution.

Known only from the type locality.

### Raveniola yajiangensis sp. n.

Taxon classificationAnimaliaAraneaeNemesiidae

http://zoobank.org/67450C7D-41B8-438E-BD34-E7FAE4FCBDB4

Figs 17 , 18 , 19

#### Type material.

Holotype ♂ – CHINA: Sichuan Province, Yajiang County, Yajiang [27°50.119'N, 99°42.426'E, elevation 3285 m], 7 June 2001, X. Yu & H. Zhang (IZCAS). Paratypes: same area but Longjiangbian [27°49.119'N, 99°41.426'E, elevation 3265 m], 27 May 2009, X. Yu & H. Zhang – 1♀ (IZCAS).

#### Etymology.

The specific epithet, a Latinised adjective, refers to the type locality.

#### Diagnosis.

**Figure 17.**
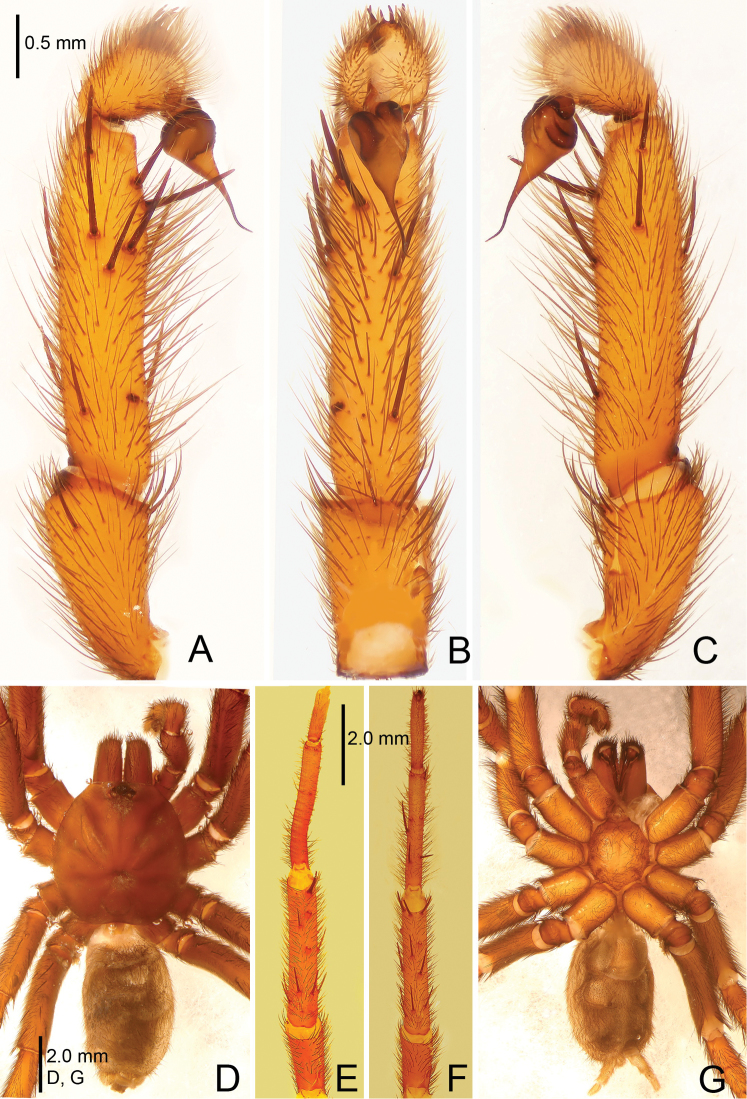
*Raveniola yajiangensis* sp. n., male holotype. **A** palp, prolateral view **B** palp, ventral view **C** palp, retrolateral view **D** habitus, dorsal view **E** leg I, ventral view **F** leg II, ventral view **G** habitus, ventral view. Scale bars: 0.5 mm (**A–C**); 2.0 mm (**D–G**).

**Figure 18.**
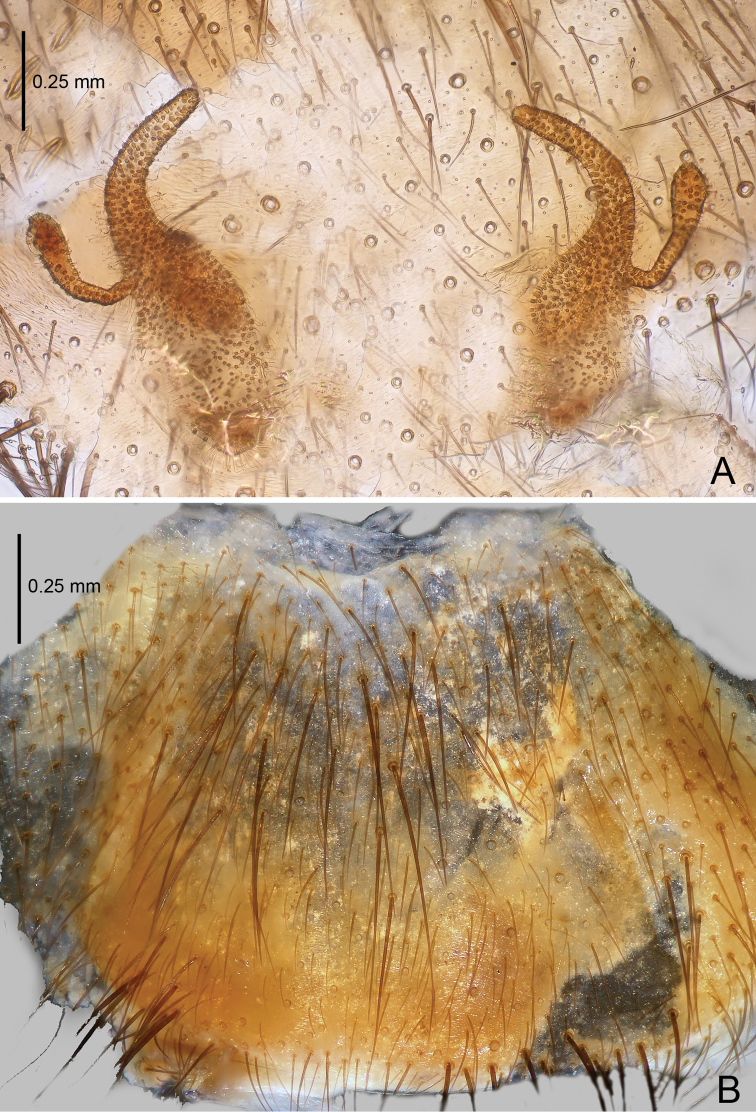
*Raveniola yajiangensis* sp. n., female paratype. **A** vulva, dorsal view **B** genital area, ventral view. Scale bars: 0.25 mm.

**Figure 19.**
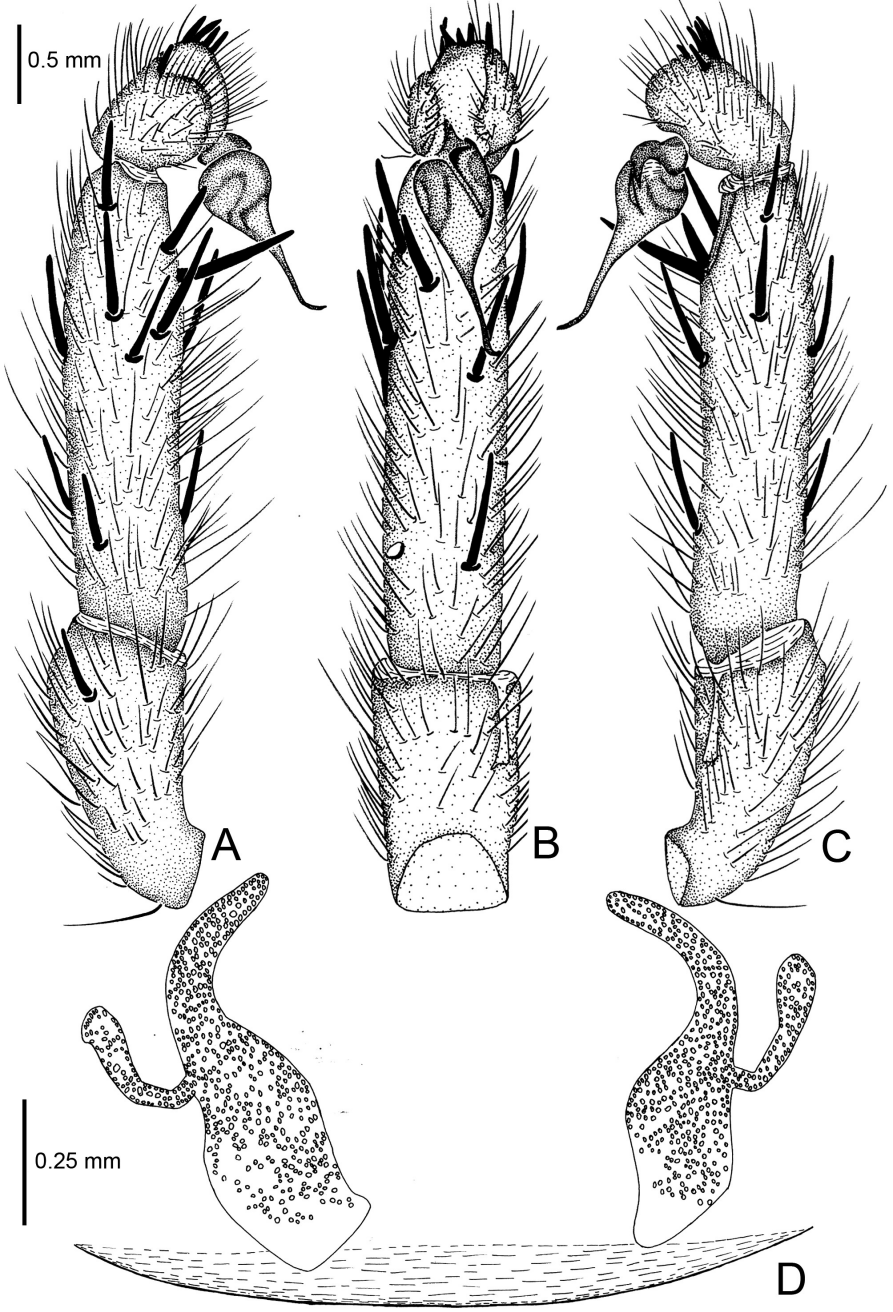
*Raveniola yajiangensis* sp. n., male holotype (**A–C**) and female paratype (**D**). **A** palp, prolateral view **B** palp, ventral view **C** palp, retrolateral view **D** vulva, dorsal view. Scale bars: 0. 5 mm (**A–C**); 0.25mm (**D**).

Judging from the shape of the bulb and the distal portion of the embolus, this new species is similar to *Raveniola shangrila* (Zonstein and Marusik 2012, figs 33, 41) but can be distinguished by the slightly curved distal portion of the embolus (Figs 17A–C, 19A–C); conspecific females possess uniquely shaped receptacles, with the inner branches curved inward (Figs 18, 19D).

#### Description.

Male (holotype): TL 14.10, CL 5.60, CW 5.45, AL 6.20, AW 3.55. Eye diameters and interdistances: AME 0.19, ALE 0.25, PME 0.16, PLE 0.14, AME–AME 0.15, AME–ALE 0.07, PME–PME 0.45, PME–PLE 0.07. Leg lengths: I: 20.96 (6.09+2.24+5.45+4.49+2.69), II: 24.23 (6.47+3.27+5.83+5.45+3.21), III: 17.76 (4.49+2.18+4.10+4.55+2.44), IV: 22.56 (5.83+2.56+4.81+6.67+2.69). Venter as shown in Fig. 17G. Prosoma, palps and legs brown. Abdomen, including spinnerets, deep grey (Fig. 17D, G). Palpal tibia long, subcylindrical; cymbium apically with five short, stout spines; bulb long and pyriform; embolus gradually tapering to a slender bent tip (Figs 17A–C, 19A–C). Small PMS present, apical segment of PLS digitiform (Fig. 17D, G).

Female (paratype): TL 16.50, CL 6.75, CW 5.80, AL 7.25, AW 5.90. Colouration and most somatic characters as in the male. Eye diameters and interdistances: AME 0.21, ALE 0.35, PME 0.17, PLE 0.23, AME–AME 0.18, AME–ALE 0.12, PME–PME 0.53, PME–PLE 0.07. Leg lengths: I: 16.05 (5.05+2.30+3.90+3.05+1.75), II: 15.15 (4.25+2.75+3.30+2.80+2.05), III: 14.45 (4.30+1.95+2.85+3.25+2.10), IV: 19.90 (5.25+2.25+4.40+5.25+2.75). Receptacles divided into a long digitiform inner branch that is bent inward and a short club-shaped outer lobe that is bent anteriad (Figs 18, 19D).

#### Distribution.

China: southern Sichuan.

### Genus Sinopesa

Taxon classificationAnimaliaAraneaeNemesiidae

Raven & Schwendinger, 1995

#### Type species.

*Sinopesa maculata* Raven & Schwendinger, 1995, by the original designation.

#### Diagnosis.

*Sinopesa*, like *Raveniola*, differs from *Hermacha* and *Entypesa* by lacking serrula and metatarsal preening combs and by possessing two enlarged retroventral spines in males and divided receptacles in females. As in members of *Raveniola*, the PMS in *Sinopesa* are reduced in size and even lost in some species – a condition which has never been observed in *Hermacha* and *Entypesa*. *Sinopesa* differs from its close relative *Raveniola* by a thin and less developed scopula and by the presence of a well-developed male intercheliceral tumescence (which is less developed in *Raveniola* and completely lost in all Chinese members of this genus).

#### Composition.

Six species – *Sinopesa chengbuensis* (Xu & Yin, 2002) (China), *Sinopesa chinensis* (Kulczyński, 1901) (China), *Sinopesa ninhbinhensis* sp. n. (Vietnam), *Sinopesa kumensis* Shimojana & Haupt, 2000 (Ryukyu Isles), *Sinopesa maculata* Raven & Schwendinger, 1995 (Thailand) and *Sinopesa sinensis* (Xu & Yin, 2002) (China). The new species is described below.

#### Key to species of *Sinopesa*

Males of *Sinopesa chengbuensis* and females of *Sinopesa ninhbinhensis* sp. n. are unknown

**Table:** 

1	Males	**2**
–	Females	**6**
2	PMS present	**3**
–	PMS absent	**5**
3	Dorsal abdominal pattern present. Palpal tibia cylindrical, embolus hooked	**4**
–	Abdomen uniformly coloured. Palpal tibia arched, embolus corkscrew-shaped (see Shimojana and Haupt 2000: fig. 3A–B)	***kumensis***
4	Large species: TL 17 mm. Embolus long: approximately half as long as palpal tibia (see Song et al. 2001: fig. 17H)	***sinensis***
–	Small species: TL 10–12 mm. Embolus short: approximately 0.3 times as long as palpal tibia (see Zonstein and Marusik 2012: fig. 46)	***chinensis***
5	Abdomen spotted; embolus corkscrew-shaped (Zonstein and Marusik 2012: figs 2 and 36)	***maculata***
–	Abdomen uniformly pale; embolus with hooked tip (Figs 20A–C, D, G, 21A–C)	***ninhbinhensis* sp. n.**
6	PMS present; abdomen uniformly coloured; receptacles U- or Y-shaped	**7**
–	PMS absent; abdomen spotted; receptacles Y-shaped, with short inner and longer outer branch (Raven and Schwendinger 1995: fig. 7G)	***maculata***
7	Receptacles U-shaped, with inner and outer branches equal in length (Xu and Yun 2002: fig. 7)	***chengbuensis***
–	Receptacles Y-shaped, with outer branch twisted and much longer than the very short inner branch (Shimojana and Haupt 2000: fig. 3F)	***kumensis***

### Sinopesa ninhbinhensis sp. n.

Taxon classificationAnimaliaAraneaeNemesiidae

http://zoobank.org/7D0D0ADA-6B9A-4BC4-8379-6AA61F590E4F

Figs 20 , 21

#### Type material.

Holotype ♂ – VIETNAM: Ninh Binh Province, disturbed forest of Cuc Phuong National Park [20°17.066'N, 105°40.253'E, elevation 273 m], pitfall traps, March 1–30, 2008. Paratypes: 26♂ (IZCAS), same data as holotype.

#### Etymology.

The specific epithet, a Latinised adjective, refers to the type locality.

#### Diagnosis.

**Figure 20.**
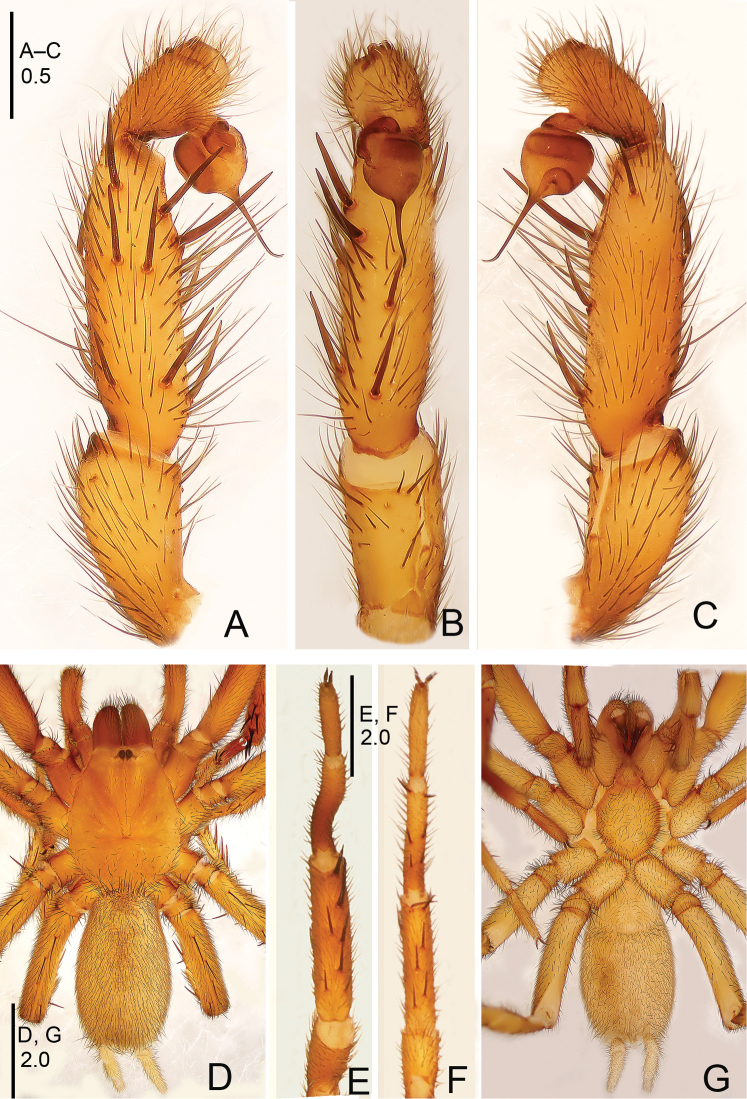
*Sinopesa ninhbinhensis* sp. n., male holotype. **A** palp, prolateral view **B** palp, ventral view **C** palp, retrolateral view **D** habitus, dorsal view **E** leg I, ventral view **F** leg II, ventral view **G** habitus, ventral view. Scale bars: 0.5 mm (**A–C**); 2.0 mm (**D–G**).

**Figure 21.**
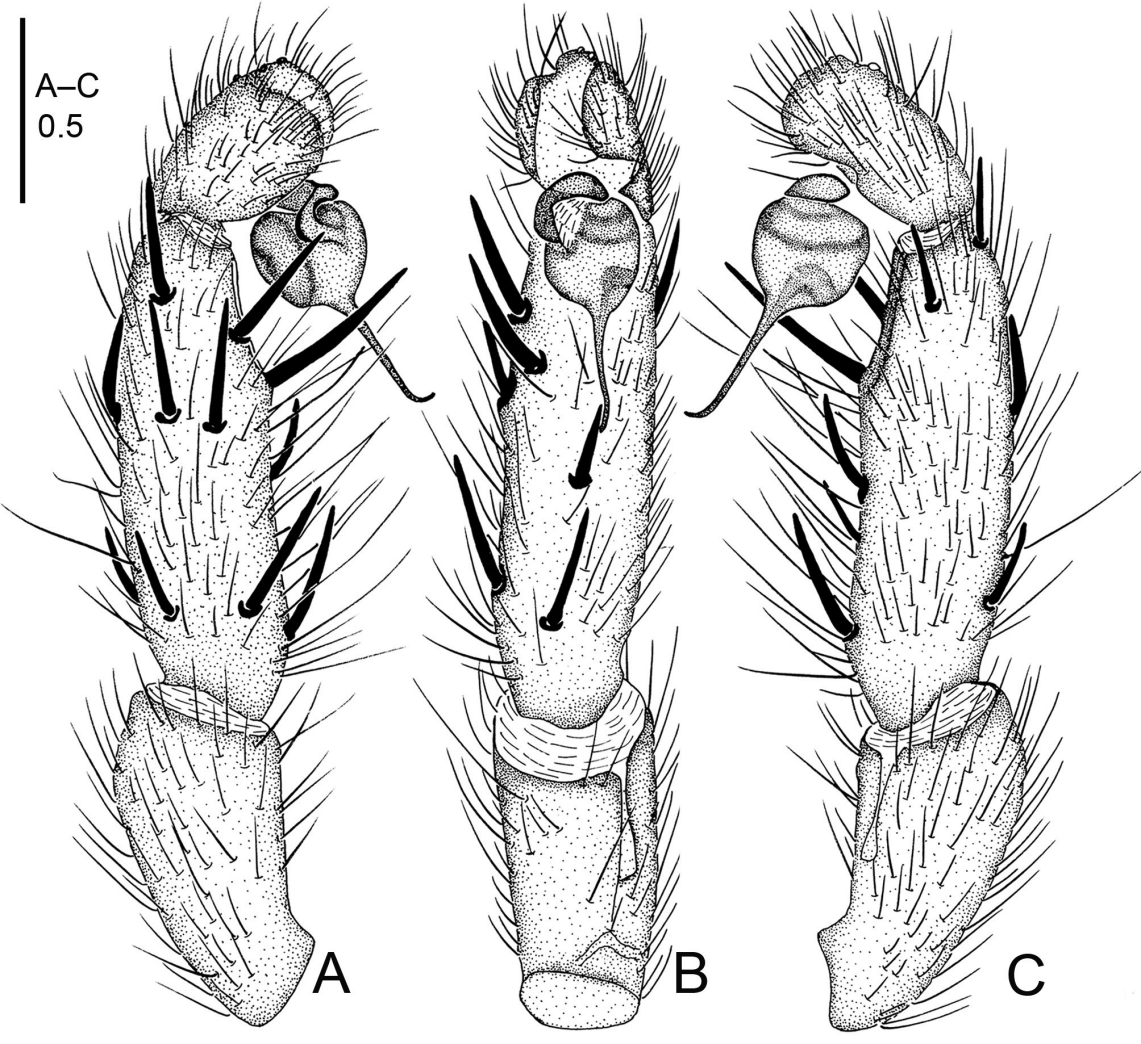
*Sinopesa ninhbinhensis* sp. n., male holotype. **A** palp, prolateral view **B** palp, ventral view **C** palp, retrolateral view. Scale bar: 0.5 mm.

The new species shares with *Sinopesa kumensis* the complete absence of an abdominal pattern and the presence of a short male palpal tibia, but it can be distinguished from the latter species by the complete absence of PMS and by the presence of a globular bulb and of a gradually tapering and apically hooked embolus (oval and corkscrew-shaped, respectively) in *Sinopesa kumensis* (see Figs 20A–C, 21A–C, and Shimojana and Haupt 2000: figs. 3A–B).

#### Description.

Male (holotype): TL 7.55, CL 2.95, CW 2.45, AL 3.20, AW 1.85. Eye diameters and interdistances: AME 0.11, ALE 0.14, PME 0.12, PLE 0.10, AME–AME 0.07, AME–ALE 0.04, PME–PME 0.26, PME–PLE 0.03. Leg lengths: I: 9.90 (2.75+1.50+2.55+2.15+1.25), II: 8.10 (2.25+1.15+2.10+1.50+1.10), III: 8.15 (1.75+0.95+2.00+2.15+1.30), IV: 10.60 (2.80+1.15+2.40+2.80+1.45). Venter as shown in Fig. 20G. Maxillae with a few (*ca.* 10) cuspules. Prosoma, palps and legs light brown. Abdomen, including spinnerets, light grey (Fig. 20D, G). Metatarsus I considerably curved and bent (Fig. 20E). PMS absent, apical segment of PLS digitiform (Fig. 20D, G). Palpal tibia moderately short and slightly swollen; bulb globular, thin and narrow-based embolus hooked distally (Figs 20A–C, 21A–C).

Female. Unknown.

#### Variation.

Total length: 6.95–7.70 (n=10).

#### Distribution.

Vietnam: Ninh Binh Province.

## Supplementary Material

XML Treatment for Nemesiidae

XML Treatment for Raveniola

XML Treatment for Raveniola alpina

XML Treatment for Raveniola bellula

XML Treatment for Raveniola chayi

XML Treatment for Raveniola gracilis

XML Treatment for Raveniola montana

XML Treatment for Raveniola rugosa

XML Treatment for Raveniola spirula

XML Treatment for Raveniola yajiangensis

XML Treatment for Sinopesa

XML Treatment for Sinopesa ninhbinhensis

## References

[B1] Li S Wang X (2015) Endemic spiders in China. Online at http://www.ChineseSpecies.com [accessed 7 July 2015]

[B2] Raven RJ Schwendinger PJ (1995) Three new mygalomorph spider genera from Thailand and China (Araneae). Memoirs of the Queensland Museum 38(2): 623–641.

[B3] Schwendinger PJ (1996) The fauna of orthognathous spiders (Araneae: Mesothelae, Mygalomorphae) in Thailand. Revue Suisse de Zoologie (Special Edition) 2: 577–584.

[B4] Shimojana M Haupt J (2000) A new nemesiid (Arachnida, Araneae) from the Ryukyu Archipelago, Japan. Zoosystema 22(4): 709–717.

[B5] Siliwal M Molur S Raven R (2015) New genus with two new species of the family Nemesiidae (Araneae: Mygalomorphae) from Arunachal Pradesh, India. Journal of Asia-Pacific Biodiversity 8: 43–48. doi: 10.1016/j.japb.2015.01.005

[B6] Song DX Zhu MS Chen J (2001) The Fauna of Hebei, China: Araneae. Hebei Science & Technology Publishing House, Shijiazhuang, 510 pp [In Chinese]

[B7] World Spider Catalog (2015) World spider catalog. Natural History Museum Bern Online at http://wsc.nmbe.ch [version 16, accessed on 7 July 2015]

[B8] Xu X Yin CM (2002) A new species of the genus *Raveniola* from Baiyundong Cave, Hunan Province (Araneae: Nemesiidae). Acta Zootaxonomica Sinica 27: 474–476.

[B9] Zhu MS Zhang F Zhang JX (1999) A new mygalomorph spider (Nemesiidae: *Raveniola*) from China. Journal of Hebei University 19: 366–368.

[B10] Zonstein SL (1987) A new genus of mygalomorph spiders of the subfamily Nemesiinae (Aranei, Nemesiidae) in the Palearctic fauna. Zoologicheskii Zhurnal 66(10): 1013–1019. [In Russian]

[B11] Zonstein S Marusik YM (2012) A review of the genus *Raveniola* (Araneae, Nemesiidae) in China, with notes on allied genera and description of four new species from Yunnan. ZooKeys 211: 71–99. doi: 10.3897/zookeys.211.3060 22930649 10.3897/zookeys.211.3060 PMC3426841

[B12] Zonstein SL Marusik YM (in press) A review of the spider genus *Atmetochilus* of Sumatra, Indonesia, with first analysis of male characters and description of three new species (Araneae, Nemesiidae). Zoological Studies. 10.6620/ZS.2016.55-10 PMC6511818 31966155

